# Exploring structural diversity across the protein universe with The Encyclopedia of Domains

**DOI:** 10.1126/science.adq4946

**Published:** 2024-11-01

**Authors:** Andy M. Lau, Nicola Bordin, Shaun M. Kandathil, Ian Sillitoe, Vaishali P. Waman, Jude Wells, Christine A. Orengo, David T. Jones

**Affiliations:** 1 Department of Computer Science, University College London, London, WC1E 6BT, UK; 2 Institute of Structural and Molecular Biology, University College London, London, WC1E 6BT, UK; 3 Centre for Artificial Intelligence, University College London, London, WC1V 6BH, UK

## Abstract

The AlphaFold Protein Structure Database (AFDB) contains over 200 million predicted protein structures, composed of domains: independently folding units found in multiple structural and functional contexts. Identifying domains can enable many functional and evolutionary analyses, but has remained challenging due to the sheer scale of the data. Using deep learning methods, we have detected and classified every domain in AFDB, producing The Encyclopedia of Domains. We detect nearly 365 million domains, over 100 million more than can be found by sequence methods, covering over 1 million taxa. Reassuringly, 80% of these domains are similar to known superfamilies, greatly expanding representation of their domain space. We uncover over 10,000 new structural interactions between superfamilies and thousands of new folds across the fold space continuum.

The AlphaFold Protein Structure Database (AFDB) (1, 2) is a groundbreaking initiative which significantly broadened the protein structure universe by expanding 3D representation to over 200 million UniProt sequences. The implications of the AFDB have been profound, not only in academic research in the life sciences but also in the commercial sphere, where the integration of novel data and the advanced technologies used to generate accurate structures is being explored for next-generation structure-based drug discovery (3).

Notwithstanding its revolutionary impact, the AFDB is not without its limitations and presents a new set of challenges. The sheer scale of the data makes many traditional tools and pipelines, originally designed for considerably smaller datasets, inadequate for navigating the extensive number of structures and sequences within. This necessitates an evolved strategy, warranting a new perspective on how best to represent and traverse the data and complex relationships within such an expansive database, as well as requiring synergy between new algorithmic methods and computational hardware. Recent studies explored AFDB by partitioning full-length AFDB models into clusters of structurally similar proteins, as well as characterising their functions (4, 5). At a more granular level, Bordin et al. (6) and Schaeffer et al. (7) interrogated the composition of specific proteomes (the initial AFDB 21 model organism dataset, and 48 proteomes, respectively), cataloguing domains under the CATH (8, 9) and ECOD (10) frameworks.

Domain discovery is possible via sequence- and structure-based approaches, with Pfam (11, 12) and Gene3D (13) as prime examples of the former. The Pfam database describes collections of protein families, each represented by a multiple sequence alignment (MSA) and a hidden Markov model (HMM) profile (11, 12). In Gene3D, sequences of existing CATH superfamilies, assigned via structure, are leveraged to discover new domains in sequence space via representative profile HMMs (13). Sequence-based discovery allows for greater coverage but is limited by HMMs detection capabilities, often failing on remote relatives. Structure-based assignments allow for higher quality domain boundaries and can reveal very remote relatives but have been limited by the low numbers of experimental structures. The task is further complicated by conceptual difficulties when performing structural comparisons, such as the lack of a statistical null model (compared to the use of random sequences for sequence comparisons).

An essential perspective yet to be explored in the context of the AFDB is the comprehensive mapping and analysis of protein domains across various branches of the Tree of Life using structural data. The relationships between protein folds and domains have been extensively highlighted by structural databases such as CATH (8, 14, 15), ECOD (10), SCOP (16) and SCOPe (17), with differences in criteria for which regions are assigned to domains in proteins. Although early comparisons across domain databases showed agreement for fold level assignments (10, 18, 19), significant differences exist for the definition of a protein domain. CATH recognises that some structures can be decomposed into further structural units, each with their own repertoire of observed variations, while SCOP takes into account the idea of fold recurrence - whether a subunit has been observed as reoccuring in another family or only as a single domain (18).

Examination of the AFDB through the lens of the CATH framework holds the potential to reveal unprecedented insights that can illuminate subtle yet important connections between structure and function across large swathes of organisms. As such, a structure-based mapping of domains within the AFDB promises not only to massively facilitate the exploration of these relationships, but also provide a foundation for further annotation.

In this study, we present a comprehensive analysis of domain composition for the entirety of the AFDB (version 4). This description encompasses over 364 million putative domains, derived from more than 214 million protein sequences across more than 1 million taxa. The identification of these domain structures is made via the consensus of three automated parsing methodologies: Merizo (20), Chainsaw (21) and UniDoc (22). Further, by employing structural comparison methods such as Foldseek (23) and an in-house deep learning method called Merizo-search, more than 251 million domains can be placed on the CATH hierarchy.

## A high-throughput strategy for identifying structural domains in the AFDB

**Fig. 1:**
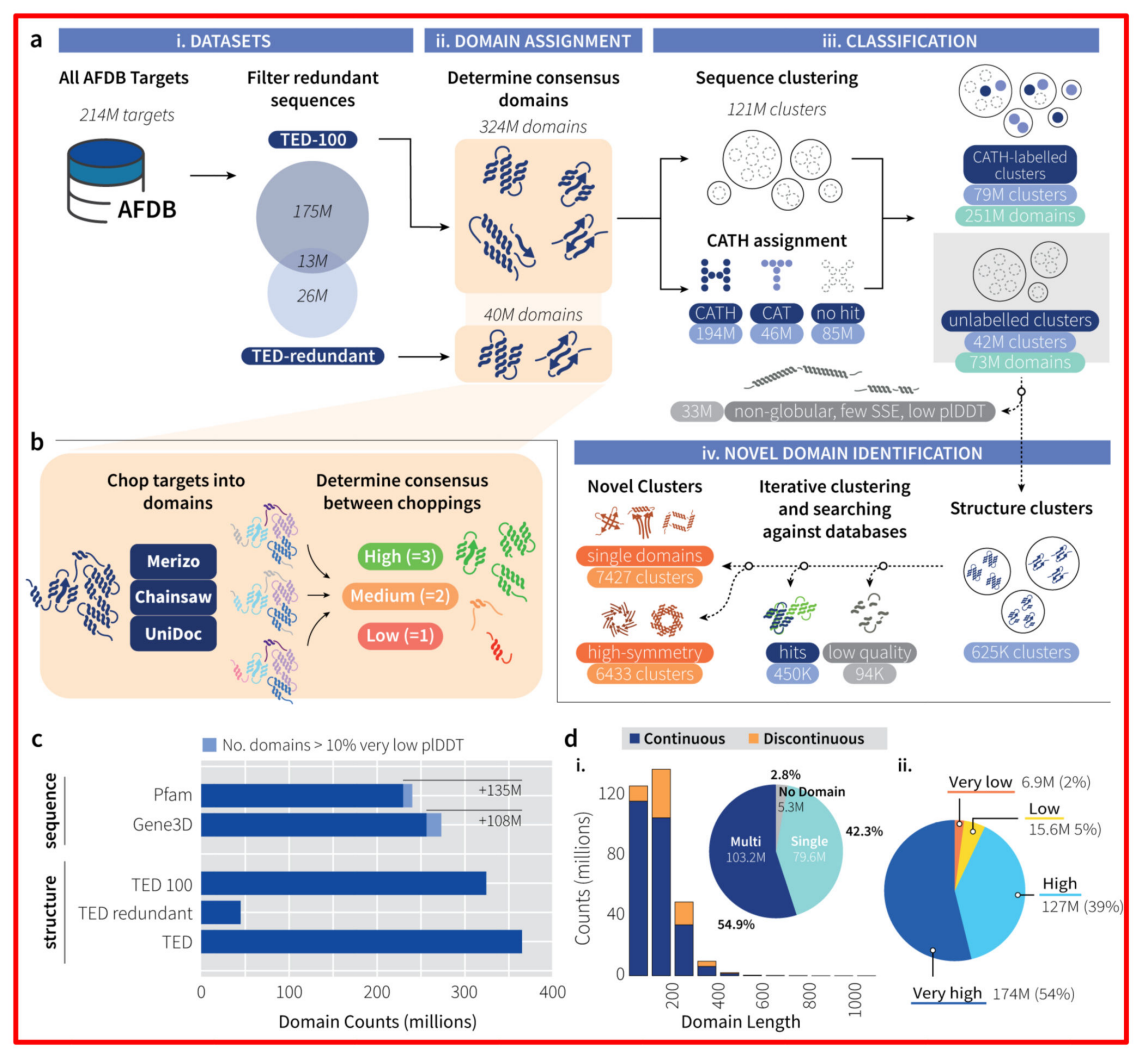
Overall workflow. (**a**) i. 214 million AFDB target sequences are filtered by 100% sequence identity in order to avoid bias. This identifies 188 million non-redundant targets (TED-100) and a set of sequence-redundant targets (TED-redundant). ii. Both TED-100 and TED-redundant undergo automated domain parsing, with assignments derived from consensus among the three methods. iii. TED-100 domains are processed by MMseqs2, creating over 121 million clusters at 50% identity. Concurrently, domains are matched to CATH domains via Foldseek and Merizo-search, categorised into superfamily (C.A.T.H), topology (C.A.T), or no-matches. Domains found by Merizo-search nearest neighbour matches are considered as topology-level matches. Clusters are annotated with CATH labels, creating partially labelled and unlabelled clusters. Low-quality domains in unlabeled clusters are filtered out. iv. Resultant domains undergo a new workflow for identification, involving clustering and database searches for matches to known structures. Poor quality domains (non-protein-like) are identified using an in-house deep learning method (Methods). Novel domains are additionally scored on internal symmetry using the SymD program (Methods). (**b**) Full-length targets are subjected to automated domain parsing by Merizo, Chainsaw and UniDoc. A consensus is taken by identifying assignments where three (high), two (medium) methods agree or no consensus is found (low). Only high and medium consensus domains are analysed further. (**c**) Comparison of domains identified by sequence (Pfam and Gene3D) versus structure-based methods (TED). The "TED" count combines TED-100 and TED-redundant. (**d**) i. Domain length distribution and proportion of identified continuous (blue) and discontinuous (orange) domains. Inset shows proportion of single, multi-domain and number of targets with no identified domains (n=188,914,411). ii. Average plDDT distribution for TED-100 domains (n=324,389,697) across confidence bins: dark blue/very high (plDDT >= 90), blue/high (90 > plDDT >= 70), yellow/low (70 > plDDT >= 50), and orange/very low (plDDT < 50).

Our workflow combines three state-of-the-art domain parsing methods together with structure classification algorithms to identify known domain folds (Fig. 1a-b; Methods) within the AFDB. Using this workflow, we identified a total of 364 million ‘TED’ domains across the AFDB (Fig. S2, Table S1) - 100 million more domains than found via sequence-based methods (Fig. 1c). TED-100 describes a roughly 42:55 division between single and multidomain proteins (Fig. 1d-i), with the latter up to 20 domains in composition (Fig. S3). Only 2.8% of targets (5.3 million) in TED-100 lack identifiable domains, compared to 33.9% (64.1 million targets) in Gene3D and 26.2% in Pfam (49.4 million targets) (Fig. S4). In TED, these targets either consist entirely of non-domain residues (NDR) or lack any consensus among the three domain segmentation methods employed. Across superkingdoms, the fraction of NDRs varies, with approximately 10% identified across archaeal and bacterial lineages and up to 30% in eukaryotes (Fig. S5).

Analysis of the average predicted local distance difference test (plDDT) scores for TED-100 reveals that the majority of domains fall within the "very high" to "high" bins, with only approximately 2% within the lowest bin (Fig. 1d-ii). Since our domain segmentation methods do not consider residue plDDT when determining domains, this suggests that our domain finding pipeline effectively identifies plausible domains within the well-folded areas of AF2 models.

## Classification of TED domains into the CATH hierarchy

The 324 million domains in TED-100 were clustered by sequence using MMseqs2 (24) and compared against CATH representative domains using fast structure searching methods (Fig. 1a-iii, Methods). This produced approximately 121 million clusters at 50% sequence identity and a minimum coverage of 90% (Fig. S6; Methods). The majority of these clusters comprise just singleton sequences (roughly 81 million), with the largest non-singleton cluster comprising 12,847 domains.

Parallel to sequence clustering, we use a combination of Foldseek (23) and Merizo-search (25) (an in-house structure search method using domain coordinate embedding) to search all TED-100 domains against CATH SSG5 domains (9) (Methods), allowing 194 million domains to be assigned with CATH superfamily (H) labels and 46 million at the topology (T) level (Methods). These labels were further validated by scanning domain sequences against an updated library of HMMs for CATH PDB domains. Approximately 171 million superfamily predictions by Foldseek could be confirmed with exact HMM superfamily matches (88.54%), with an additional 1.8 million domains (0.95%) confirmable at the fold level. Only 4.1 million of the 16 million Foldseek predictions for fold matches on CATH can be validated by HMM scans (25.8%), with 11.8 million fold predictions and 20.3 million superfamily predictions by Foldseek not confirmed by an HMM match, suggesting an expansion by 15.4% in CATH labelled domain coverage using AFDB structures over HMM-based sequence assignments.

By identifying sequence clusters with any CATH label assigned domains, the clusters were partitioned into two categories: 78 million clusters (over 251 million domains, nearly 80% of all TED-100 domains) which contain at least one CATH-labelled member, spanning 148 million proteins, and 26 million proteins having no domain annotations in Pfam and 30 million having none in Gene3D (Fig. S7). The remaining 41 million clusters have no members with CATH labels (approximately 73 million domains; Fig. S6). The absence of similarity to CATH domains in the latter clusters could be attributed to them being novel folds, extremely divergent relatives of existing CATH domains or simply being incorrect models, making them unmatchable to known folds.

## Enrichment of Fold Representation by TED

**Fig. 2:**
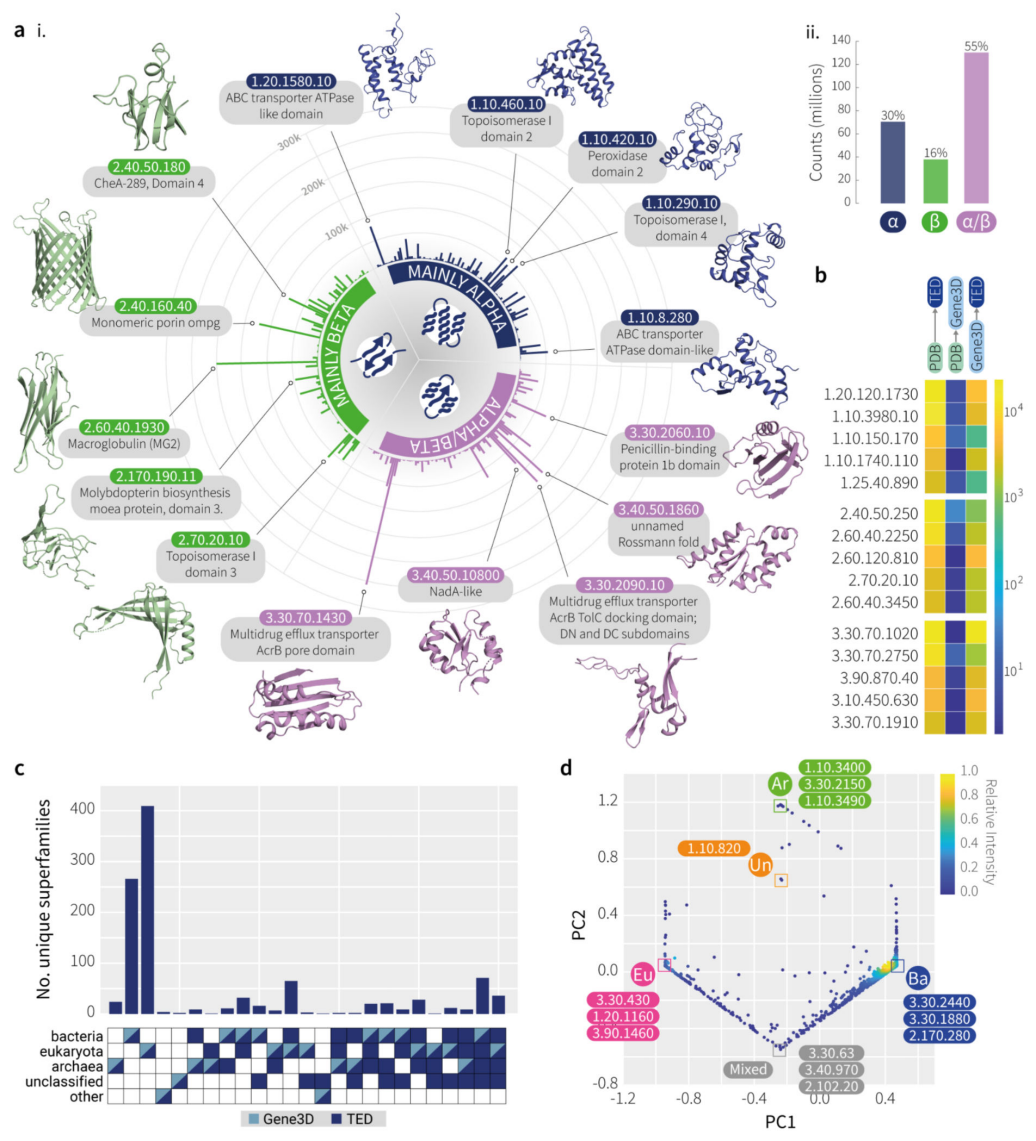
Classification of TED domains using the CATH hierarchy. **(a)** i. The top 100 superfamilies in TED-100 for each CATH class where more matches to CATH superfamilies have been identified via structural hits in TED, compared to sequence hits in Gene3D. ii. Proportion of domains matched to CATH classes (n=238,569,631). **(b)** Enrichment of superfamily representation in TED-100 compared to PDB and Gene3D. The top 5 superfamilies of each CATH class are shown, where enrichment in TED-100 compared to PDB is the greatest. Colour scale represents fold-change in superfamily representation in PDB and Gene3D compared to Gene3D and TED. A full list of fold names corresponding to the CATH superfamily codes can be found in Table S2. (**c**) Expansion of CATH superfamilies to new superkingdoms in TED. Plot shows the number of unique superfamilies found in each superkingdom (across the 653,460 taxa of TED-100) according to Gene3D and TED assignments. Each column along the horizontal axis depicts the number of superfamilies that are exclusive to a single superkingdom when only considering Gene3D assignments, but are expanded into one, two or three additional superkingdoms in TED. Only superfamilies where Gene3D domains are exclusive to a single superkingdom are shown (n=1061). **(d)** Exclusivity of CATH topologies across superkingdoms. PCA of normalised CATH topology counts across five superkingdoms: eukarya (Eu), bacteria (Ba), archaea (Ar), unclassified and other sequences (Un). The ‘mixed’ category comprises topologies found in roughly equal proportions in Eu/Ba domains. Examples of superkingdom-exclusive topologies are shown for each category.

To develop an understanding of how folds are distributed across the AFDB, we assessed the composition of TED using the CATH hierarchy. Fig. 2a shows the top 100 CATH superfamilies of each class (alpha, beta and alpha/beta), which are greatly enriched in TED-100 compared to baseline sequence hits in Gene3D.

These folds and architectures are unevenly distributed across the Tree of Life, however the majority of folds (61%) are shared and reused across all superkingdoms, suggesting essential roles for cellular life. Some folds were found across two superkingdoms (18.5%), while others are more exclusive, with 0.5%, 9% and 11% of CATH folds found only in archaea, eukarya and bacteria, respectively.

The most abundant superfamilies in TED-100 and CATH are shown in Fig. S8 and Fig. S9. Directly comparing the top superfamilies assigned in TED compared to CATH, in terms of raw domain counts, sees the promotion of several superfamilies into the top 5 of each class, including the MFS general substrate transporter-like domain, translation factors and the FAD/NAD(P)-binding domain (Fig. S8). The set of superfamilies highly enriched in TED include those associated with the archetypal multi-drug efflux pump AcrB. AcrB is a Resistance-Nodulation-Division (RND) transporter and forms part of the AcrAB-TolC efflux pump in bacteria where it is responsible for the export of harmful substances such as antibiotics, contributing to antibiotic resistance (26). The constituent domains of AcrB, including the pore domain, transmembrane domain and TolC docking domain, are found greatly enriched in TED compared to the PDB, providing up to nearly a thousand-fold increase in representation for these biologically important superfamilies.

The pore domain, which forms part of the pore selectivity filter in RND transporters, is principally found in bacterial species and in a small minority of archaeal and eukaryotic lineages based on Gene3D hits. However, structure-based searching via TED expands the coverage of the pore domain superfamily into an additional 18 archaeal, 1315 bacterial and 284 eukaryotic lineages unique to TED, which evaded even HMM searches. This broader coverage of organisms, revealed by structural comparisons, may reveal potential evolutionary events, such as lateral gene transfer between bacteria and eukaryotes. This expansion is exemplified in Fig. 2c, where we show that over 1000 CATH superfamilies, described by Gene3D sequence matches as occurring solely in a single superkingdom, could actually be mapped to other branches when structure was taken into account in TED.

TED also enables us to better study the distribution of folds across different branches of the tree of life. Among the 193 million TED domains with superfamily labels, we observed folds that were exclusively localised to specific superkingdoms. These findings are shown in Fig. 2d, where folds are summarised at the CATH topology level, visualised by principal component analysis (PCA), and where points at the vertices represent exclusive occurrences of a topology and all its superfamilies, in a particular superkingdom.

## Novel high-symmetry architectures

From our TED workflow (Fig. 1), we identified 41 million sequence clusters which could not be linked to CATH superfamilies. Representatives of these clusters were subjected to a workflow aimed at identifying novel domain folds (Fig. 1a-iv; Methods).

While reviewing these clusters, it was apparent that we would need to treat repeat architectures with high internal symmetry separately. A good example of domains in this class are the various WD40 beta propellers, which are considered distinct domain architectures in their own right, but clearly comprise repeats of domain-like units. To identify similar domains in our workflow, we calculated Z-scores using the SymD program (27), sequestering any cluster representatives with a symmetry Z-score of greater than 9, into a new category of 6,433 highly symmetric novel fold clusters (Fig. S10).

**Fig. 3:**
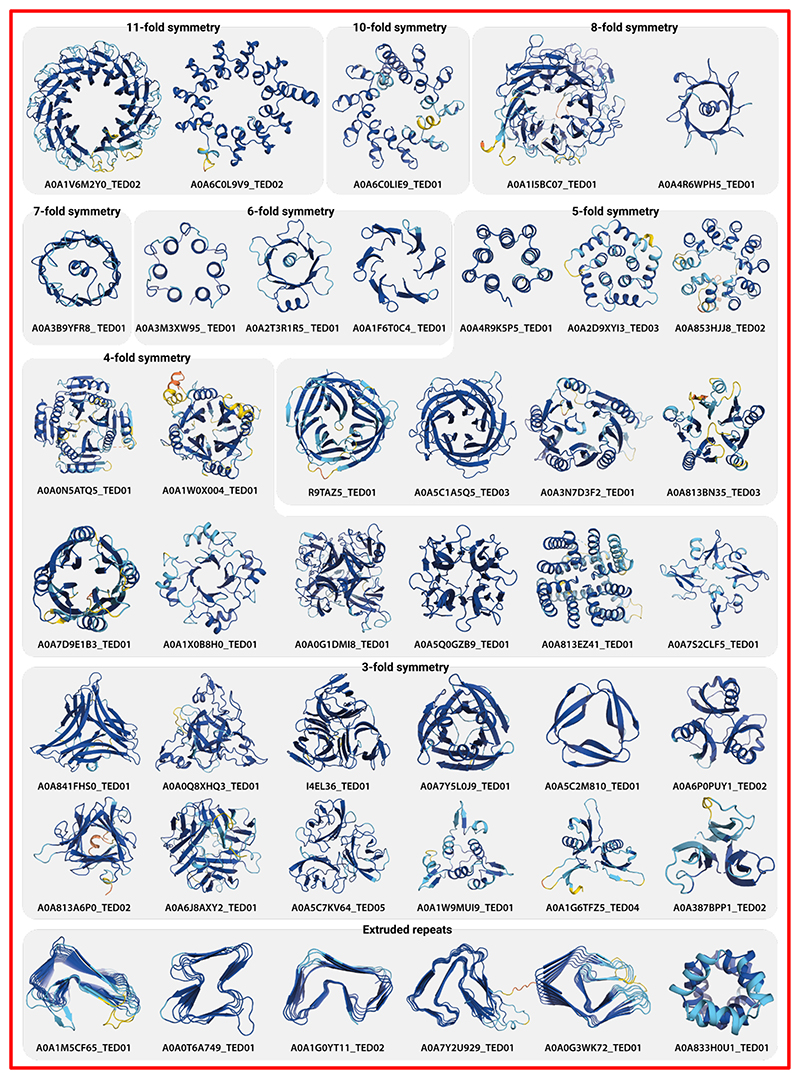
Examples of high-symmetry domains and extruded repeats. Domains are identified as part of the novel domain identification pipeline and are identified as domains with high internal symmetry via scoring with the SymD program (Methods). Extruded repeats are domains with a high number of ordered cyclical repeats projecting along one axis. Colouration follows plDDT confidence bins as per the AFDB (dark blue/very high: plDDT >= 90, blue/high: 90 > plDDT >= 70, yellow/low: 70 > plDDT >= 50 and orange/very low: plDDT < 50).

Within these clusters, we find new architectures such as an 11-bladed beta-propeller, a closed alpha ring-like 11-helix propeller and 6-helix propeller which have not been seen before (Fig. 3). Various other propeller arrangements including the “beta-flower” domain shown by Durairaj et al. (4), were also identified, with some visually striking examples shown in Fig. 3.

More curiously, we find a broad category of architectures which are composed of cyclic repeats, extruded along an axis to form highly repetitive and symmetric structures which we call “extruded repeats”. Several studies (28, 29) and databases such as the Database of Structural Repeats in Proteins (DbStRiPs) (30) have curated repeat architectures from the PDB, including alpha- and beta-solenoids, and horseshoes formed of alpha-repeats such as HEAT, ankyrin and armadillo. Fig. 3 showcases some complex examples of these structures, with some featuring highly varied and unstructured loops between repeating units (Fig. S11). Many of these domains architectures resemble other solenoid folds that were recently reported in a systematic study of beta-solenoid fold space (29).

## Novel Domains and their distribution across the Tree of Life

The remaining low-symmetry clusters were assessed on domain quality, using a variant of the Foldclass network (25) trained to identify poor quality domain choppings (Supp. Methods), and novelty by further matching against known structure libraries. The final output of our workflow produced 7,427 clusters of putative domains which appear to be well-folded but dissimilar to any known domain fold. Although there is no exact boundary between novel and just highly divergent examples of known folds, by applying a density-based anomaly detection algorithm (Methods), we could at least rank these domains by novelty relative to known domains.

**Fig. 4:**
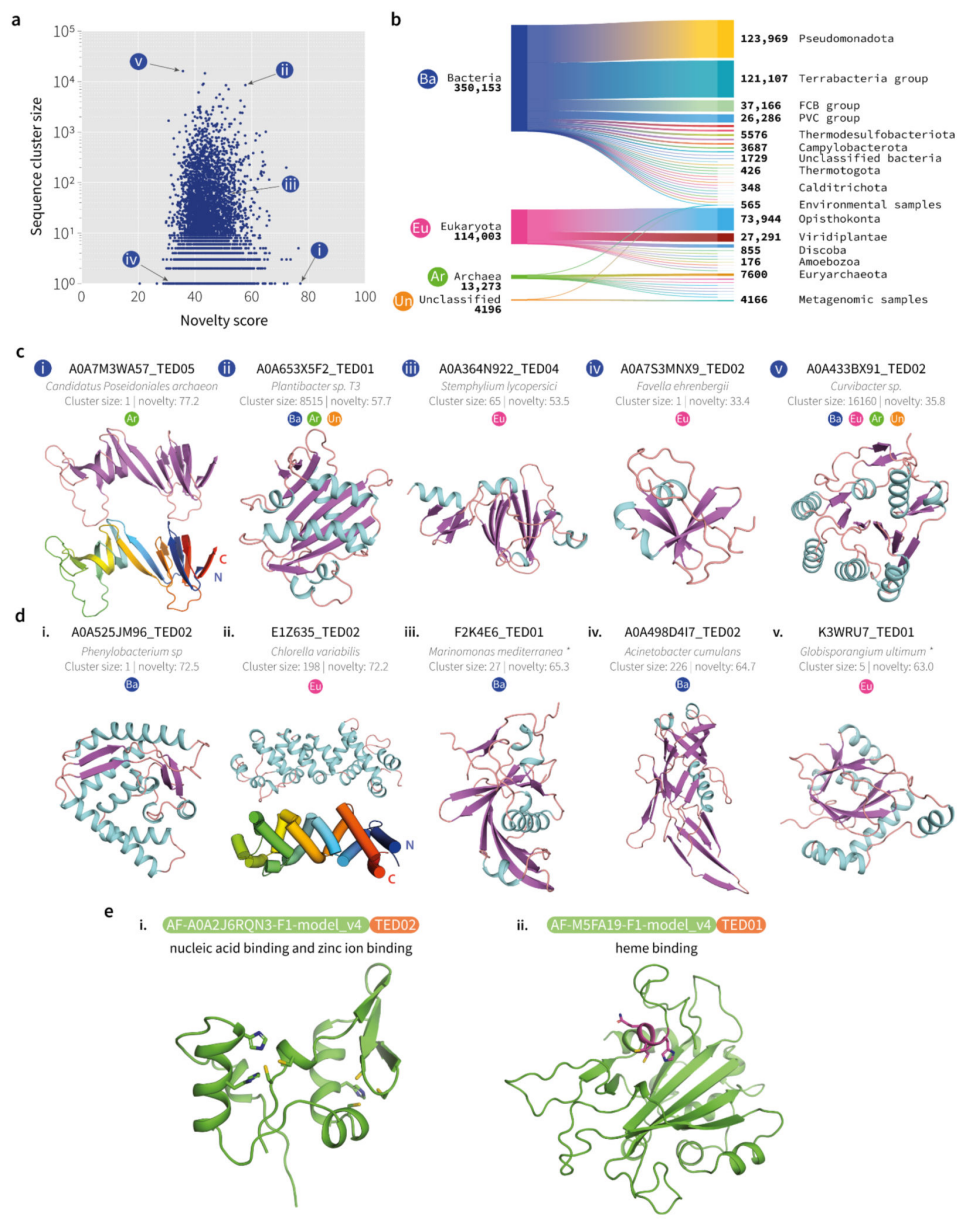
Examples of novel domain clusters identified in TED. (**a**) Comparison of domain novelty score versus sequence cluster size (n=7427). Novelty scores are predicted by the Foldclass algorithm where novel domains are ranked with a score close to 100. (**b**) Taxonomic distribution of novel domain clusters (for all sequence cluster members; n=483,732). Largest common phyla are shown across superkingdoms along with the number of domains in sequence clusters assigned to each level of the hierarchy. (**c**) Subpanels i-v correspond to labels shown in panel (a). In panel i, the bottom sub-panel shows the arrangement of strands that form the coiled hairpin loop from the N-terminus (blue) to C-terminus (red). The quoted cluster size represents the number of identified homologues at the sequence cluster level. Labels denoting superkingdoms correspond to panel (b) and represent the superkingdom that all cluster members belong to. The cluster is distributed across multiple superkingdoms when multiple labels are shown. (**d**) Examples of high-novelty structures. In panel ii, the bottom sub-panel shows the arrangement of helices that form the coiled hairpin loop from the N-terminus (blue) to C-terminus (red). Asterisks denote where organism names have been shortened: iii. *Marinomonas mediterranea* (strain ATCC 700492 / JCM 21426 / NBRC 103028 / MMB-1), v. *Globisporangium ultimum* (strain ATCC 200006 / CBS 805.95 / DAOM BR144) (*Pythium ultimum*). (**e**) Novel folds with predicted functions. i. Example of a domain predicted to have nucleic acid and zinc binding properties. Potential zinc binding site residues are highlighted as sticks. The left-hand site is composed of 2 Cys and 2 His residues, whereas the right-hand site has 3 Cys and 1 His in a tetrahedral arrangement. ii. Example of a heme binding domain. The residues of the heme *c* binding motif are highlighted.

Fig. 4 showcases examples of novel domains identified by our workflow. Over a quarter of clusters corresponded to singletons at the sequence cluster level (1930 domains), which are uniformly distributed in terms of the Foldclass novelty score. Most novel domains identified are from bacterial proteins, which is unsurprising given that the latter comprise most proteins in the AFDB and in our TED-100 dataset. Overall, these domains are distributed evenly across different phyla, but several bacterial phyla were found overrepresented when compared to baseline counts across all domains in TED-100, specifically in the PVC group, Myxococcota, Spirochaetota, Bdellovibrionota, Nitrospinae/Tectomicrobia group and Calditrichota (Fig. S12), suggesting that species within these phyla may be underrepresented in terms of domain coverage in the current PDB.

Ranked first by novelty, is a curious archaeal domain found as a sequence singleton from *Candiatus Poseidoniales archaeon* (TED: A0A7M3WA57_TED05; novelty score: 77.2; Fig. 4c-i). This protein is not documented in InterPro and no GO terms are available. The structure is composed of paired beta-strands, in a closed, twisted hairpin with both termini adjacent to one another. Reviewing the context of the full-chain from the AFDB shows that the domain forms part of an extended loop, protruding from the middle of a immunoglobulin-like domain (TED: A0A7M3WA57_TED04). The hairpin topology of A0A7M3WA57_TED05 is mirrored by another identified novel domain represented by E1Z635_TED02 shown in Fig. 4d-ii, but is alpha-helical in nature. This domain is found only in eukaryotes, primarily in species belonging to the Viridiplantae phyla (97 species) but also in a minority of Opisthokonta (14 species) and Amoebozoa (1 species), suggesting an evolutionary link between these lineages.

## Sequence-based function prediction for novel fold and repeat domains

To see if any functions could be assigned to the domains with novel folds and repeats, we use a sequence-based deep learning model (Supp. Methods) to predict Gene Ontology (GO) terms. This analysis shows that 1321/7427 (18%) of the domains in the putative novel fold set, and 1419/6433 (22%) of the repeat set can be assigned high confidence (*p* < 10^-4^) Molecular Function GO term labels. The top 20 GO terms predicted for the two sets of domains are shown in Table S4 and Table S5. Manual inspection of the domains predicted to have zinc binding and nucleic acid binding functions reveals that many of the domains contain plausible zinc binding sites (31), most containing 2 Cys and 2 His residues arranged in tetrahedral fashion, including as part of zinc finger-like supersecondary structure motifs. Fig. 4e-i shows one example containing two zinc binding sites and a possible nucleic acid binding alpha-helix, but which lacks the canonical zinc finger supersecondary motif. We also consider the set of domains predicted to have heme binding properties and find that most of these contain the canonical heme *c* binding motif (CXXCH) (32). Inspection of the three-dimensional structures reveals that each of these domains has one or more heme binding sites in a plausible conformation; one example is shown in Fig. 4e-ii, with the residues of the heme *c* binding motif highlighted. The His residue that binds the heme iron is in a conformation compatible with placement of the heme group in the pocket, which is primarily hydrophobic. The presence of clear sequence motifs and structural features consistent with the assigned functions suggests that these novel domains may indeed have the predicted functions, and further work is needed to validate the remainder of the predicted functions for these novel domains.

## Novel Interactions Between Domain Pairs

**Fig. 5:**
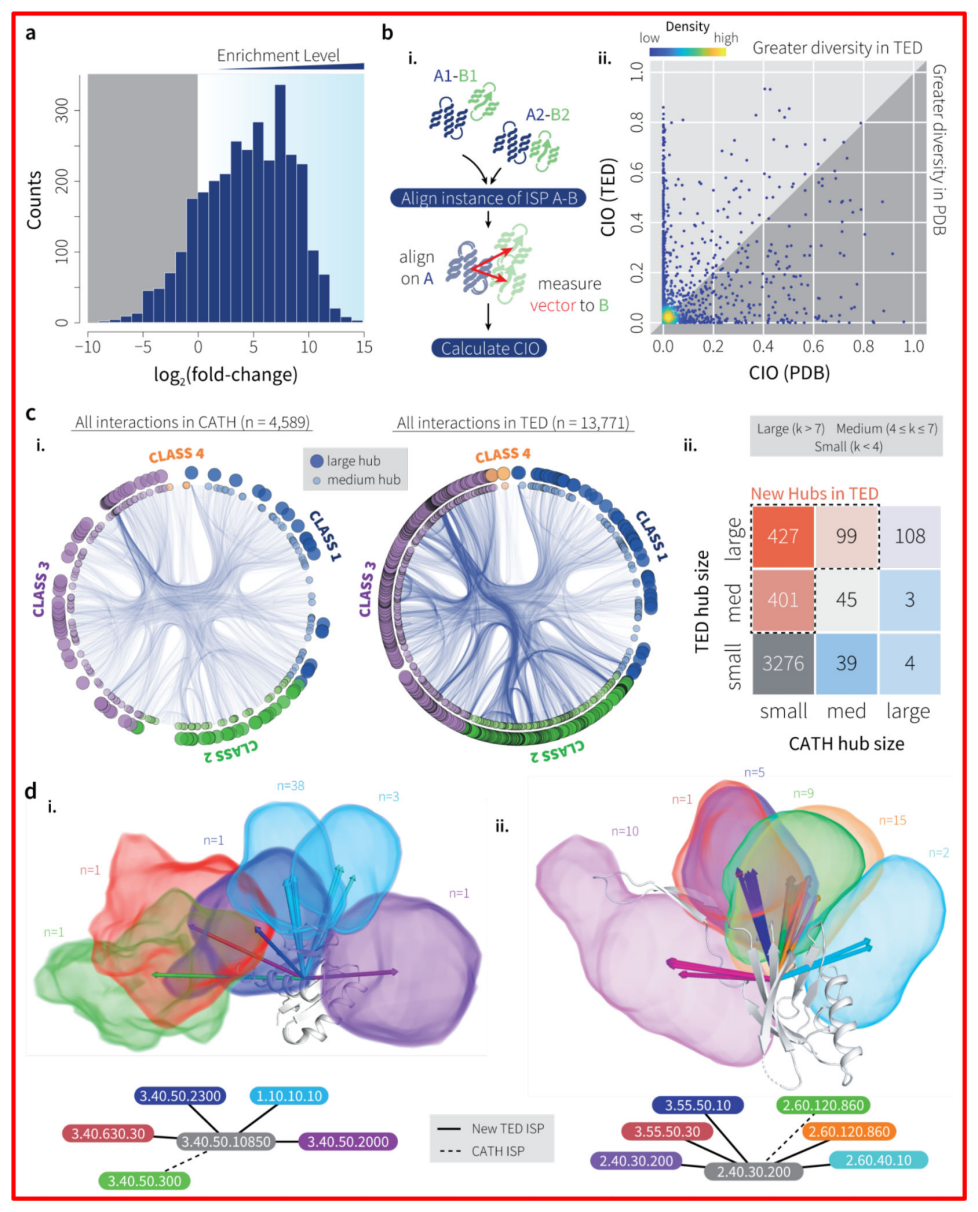
Interacting superfamily pairs (ISPs). (**a**) Enrichment of the number of instances of ISPs common to the CATH and TED datasets, expressed as log_2_(fold change) (n=3070). (**b**) i. Alignment procedure used to compute CIO values for an ISP. One domain in each instance of each ISP is used as a reference and aligned to a designated ‘master’ reference domain structure. The rotation and translation from each alignment is applied to the second, ‘tag-along’ domain to bring all domain pairs into a common frame. Vectors are then computed between the centres of mass of each pair of domains, and used to compute the CIO measure (see Methods). ii. Comparison of CIO values for ISPs common to CATH and TED. Most ISPs show a high degree of conservation in interaction patterns. (**c**) i. Hierarchical edge bundling plots illustrating differences in domain superfamily interaction patterns between CATH (left) and TED (right). Curves in the plots connect interacting superfamilies. Hubs are marked by medium (4-7 connections) and large circles (>7 connections) on the outer rim. ii. Comparison of hub domains in CATH and TED. The heatmap compares CATH superfamilies in CATH and TED as hubs, categorised as small (<4 connections), medium (5-7), and large (≥8). Hub thresholds used are from Ekman et al. (33). (**d**) Two examples of new hub superfamilies in TED, with groups of domains for interacting superfamilies placed in a common frame and represented as volumes, alongside chains involved in each group and a graph representation of each hub. The sets of interactions for superfamily 3.40.50.10850 (NtrC-like protein domain) are disjoint between CATH and TED, whereas the set of TED interactions for superfamily 2.40.30.200 (Distal tail protein domain) include that seen in CATH (orange and green). A decomposed view of d.i. appears in Fig. S15.

Unlike sequence-based domain annotations such as Gene3D, the availability of full-chain AF2 models for multidomain proteins allows us the unique opportunity to interrogate and compare domain pair packing interactions in TED and CATH. TED contains a total of 27,280,057 instances of interacting domains categorised into 13,771 Interacting Superfamily Pairs (ISPs). In contrast, the set of interacting domains in CATH consists of just 196,234 instances across 5,111 ISPs. The relative enrichment in the number of instances of ISPs common to CATH and TED is shown in Fig. 5a, which shows that most of these ISPs have many more members in TED. Assessing the diversity of interaction geometry for ISPs common to TED and CATH (Methods and Fig. 5b-i-ii), we find that for most ISPs, the diversity of interaction geometries in TED is consistent with that seen in CATH, indicating that on average, AF2 tends to recapitulate inter-domain geometries already seen in the PDB.

A small proportion (5.4%) of these ISPs show enhanced diversity in interaction geometries in TED, as measured by an increase in Conservation of Interaction Orientation (CIO) score (Methods) of 0.3 or more. A smaller proportion of ISPs (2.3%) are more diverse in CATH. We find no strong dependence of difference in CIO value on whether the ISP in question is homotypic (2 copies of domains from the same superfamily) or heterotypic (Welch two-sample *t*-test, *p*=0.141). Most of the interacting superfamily pairs (ISPs) in the TED set (10,701 out of 13,771) are unique to TED, i.e. they are not observed in CATH, and CATH contains 2,041 ISPs not seen in TED (Fig. S13).

That some interactions in CATH are not captured in TED is not entirely surprising, firstly due to the possibility of domain parsing and classification errors. Secondly, TED excludes any sequence not modelled in the AFDB; these include sequences from viruses (around 5,304,757 sequences), and longer sequences, which we estimate contain between 50 and 61 million domains (Supplementary Methods). The AFDB will also not contain experimental constructs comprising domain combinations not observed in natural sequences. Lastly, instances of ISPs in TED are filtered based on whether the constituent domains are in contact in the full-length AF2 structure, and whether they have a favourable inter-domain Predicted Aligned Error (PAE; Methods), both of which depend on the availability of homologous sequences and template structures that ideally span both domains. For these reasons, we do not expect that TED ISPs will be a strict superset of CATH ones. Nevertheless, we find that TED essentially doubles the set of known domain interactions at the superfamily level (10,701 novel TED interactions versus 5,111 known interactions in CATH).

A visual illustration of ISP sets can be seen in Fig. 5c-i, in which a path is drawn between two superfamilies if at least one interaction is observed between domains assigned to those superfamilies. This representation uses the CATH hierarchy as a guide to ‘bundle’ paths drawn between superfamilies in related parts of the CATH hierarchy (Methods; Fig. S13), and shows visually that a very large number of new interactions are seen, especially between superfamilies in CATH classes 2 and 3 (all-beta and alpha-beta classes, respectively). As mentioned above, the majority of the interactions seen in the TED set are unique to TED, and a visual comparison of the set of TED-unique interactions to all known interactions in CATH (Fig. S13) reveals a huge expansion in the set of known interactions. The network of superfamily interactions also allows us to identify superfamilies that can be considered hubs on account of their ability to interact with many other superfamilies. As shown in Fig. 5c-i-ii, a large number of superfamilies in CATH have their hub status ‘promoted’ due to new interactions seen in TED. In Fig. 5d-i-ii we show two examples of superfamilies that have been promoted in this way. Notably, the set of interactions observed in TED for these superfamilies show that they can contain only novel (in the case of 3.40.50.10850) or, in some cases, a mixture of novel and already-observed interaction modes (for 2.40.30.200).

The interaction data shows that TED greatly expands the set of known interactions and interaction modes between domains. Future work will aim to delve deeper into the interaction data and determine the functional roles that these interactions (particularly those not seen before) might play in cellular processes, in particular, taking into account the multi-domain architecture of individual proteins and their evolutionary relationships.

## Structures of redundant sequences in the AFDB

Of the 214 million structures in the AFDB, nearly 39 million are exact sequence duplicates of other proteins in the database (13 million unique sequences within this set). The 26 million redundant proteins comprise our TED-redundant set (Methods).

Remarkably, the AFDB models for these sequence-redundant proteins often diverge from one another, with approximately 42% of clusters (5.6 million) showing a maximum cluster RMSD of greater than 1Å (Fig. S16). The very largest RMSDs in the distribution tend to relate to changes in domain packing, but even at the domain fold level, changes can be observed. Fig. S16 shows the distribution of maximum pairwise RMSD for each cluster of identical sequences. We found structural variation at the chain-level (Fig. S16a), as well as in the PAE maps generated by AF2 (Fig. S16a-ii).

One explanation for our observations here is that we are looking at alternative conformers of the protein chains relating to different MSAs. However, in these cases, the input MSAs should be identical given the described modelling protocol. The larger differences are also far too large to attribute to the short relaxation step that follows the model prediction step. This is most evident in the example shown in Fig. S16a-ii, where two AFDB models for identical sequences deviate by nearly 65Å and show clear differences in the PAE map.

To further investigate structural diversity within these sequence-redundant clusters, we subjected the TED-redundant targets to our domain parsing workflow, identifying approximately 40 million domains across the set. In addition, we also derived a consensus across all structures in each identical sequence cluster (Methods). This allowed us to investigate domain-level changes in conformation between identical sequences, and identified many cases where the consensus domains were dramatically different (Fig. S16b).

## Discussion and Conclusions

What we have shown so far in developing TED is a way in which structural data in the AFDB can be augmented, by carefully breaking down structures into their component domains, allowing them to be classified through the CATH framework. This initiative not only drives forward the associations that we can make between structure and function, but as shown in our study, can be used to discover and reclaim the dark areas of fold space that are not accessible to sequence-based discovery.

Comparing TED with a recent study on the 21 model organisms dataset (6) (released prior to the 214 million release), already shows that the TED workflow identifies not only a greater number of domains, but that the domains are also of higher quality and capture many more remote homologies (Table S3). TED currently annotates domains for over 1 million taxa, of which 600,000 are currently mapped to CATH domains in TED-100, and such an extended mapping of domains to superfamilies will enable many more evolutionary discoveries. A good example of how such expansion of CATH superfamilies enhances understanding of evolutionary processes and aids inheritance of functional properties, even towards drug repurposing is shown in Fig. S17.

The coverage of TED dwarfs sequence-based assignment methods (namely Gene3D and Pfam), identifying over 100 million more domains in the AFDB compared to the latter. The proportion of proteins that each method can find domains within is shown in Fig. S4, which illustrates the advantage of considering structural domains (TED finds domains in 40-50 million proteins that Gene3D and Pfam cannot). Interestingly, prior studies based purely on sequence comparisons have suggested that between 40-65% of prokaryotic proteins are composed of multiple domains, with a higher proportion proposed in eukaryotes (34–36).

These values are comparable to those seen in TED, where we find a roughly 42:55% split between single and multidomain proteins in the AFDB (Fig. S4). Compared to TED, the proportion of multidomain proteins is much lower in Gene3D (29%) and Pfam (24%) assignments (Fig. S4).

Although most structures in the AFDB are undeniably of high quality, the sheer scale of the data means that errors and anomalies are inevitable, and these should be discussed in order to assess the overall robustness of the data. Some limitations have already been pointed out by other studies (37, 38). One such idiosyncrasy that appeared during our development of TED was the observation that models of 100% identical sequences were sometimes dramatically different. To reframe the issue, the implication of this is that for a given protein, a user may be able to find a better model or domain within the AFDB (and one that AF2 clearly places higher confidence in) if sequence-redundant copies are considered. This may mean that some alternative structures could only be detected when redundant copies are available to compare against. Given that the vast majority of AFDB entries (175/214 million) do not have duplicates, the implication here is that an unknown (but probably large) number of low-quality structures or regions in AFDB might be improvable by resampling the input MSAs and reassessing the quality of the models. Even though this might be too heavy a task to do across the whole of AFDB, users of the database should certainly bear this in mind when looking at specific domains of interest.

The obvious explanation for the existence of divergent models for a given sequence must be that the models were generated using significantly different MSA information, and we note that explicit pre-sampling of the MSAs given to AF2 has been explored recently as a way to persuade the network to generate alternate conformations (39, 40). As the structure generation pipeline in AF2 includes possible random MSA resampling during the forward pass, it could be that a borderline MSA could result in very divergent predictions on different runs due to this resampling (fixed random seeds were not used in making the AFDB). Another possibility is that the sequence data banks used for different instances of the same target sequence were inadvertently updated to newer versions if they were predicted at later times. We were not able to investigate these possibilities further ourselves as there is no information on the MSAs provided in the AFDB data, but we do suggest that it is a topic worthy of further investigation by the AFDB developers.

Fortunately, we find many examples that reiterate the notion that plDDT is an invaluable discriminator of the confidence that a user should place on a model. Fig. S18 shows an example whereby an AFDB entry has been generated within the very low confidence bin. This model features a significant number of structural defects not typical of AF2 models. “Re-folding” the sequence in ColabFold (41) produces a number of visually striking structures across the five AF2 models, the variation of which, along with their unusually low plDDT scores, strongly indicates that AF2 has hallucinated these folds and that they should likely be disregarded.

However, overall, the large proportion of domains mapping to CATH evolutionary families gives confidence in the quality of the models, capturing well the structural features of folds and preserving their distinctive structural characteristics. In this context, the performance of the domain segmentation algorithms deployed here has also been key, as early pilot work on the 21 model organisms suggested a much higher proportion of problematic AF2 models largely caused by the poor segmentation of full-length proteins into domains by the sequence-based methods. As well as confirming earlier hypotheses that the majority of domain structural families had already been characterised experimentally (42, 43), our study has also revealed some intriguing and beautiful new domain architectures and folds, especially some highly symmetric repetitive structures. Future development of TED will aim to comprehensively analyse these new folds and incorporate them into the CATH hierarchy, which will require extensive manual curation. Additionally, we recognize the need to optimise the current TED workflow for better detection of repeat structures, such as the different extruded repeats highlighted in our work. This optimisation will necessitate the development and integration of new tools and analysis methods specifically designed to identify these types of structures.

Throughout our study, we had to make a number of algorithmic decisions which were primarily motivated by the monumental amount of data we had to process in the AFDB. As such, we intend for TED to be an ongoing development, which will evolve as the data and the needs of its users do. Our aim is to provide the community with the most comprehensive summary and breakdown of the structures within the AFDB. We expect TED to be used as a starting point for a whole host of analyses, including providing a comprehensive dataset to train and test a new generation of deep learning based applications in structural biology.

## Supplementary Material

Supplementary

## Data Availability

The Encyclopedia of Domains (TED) structural domain assignments for AlphaFold Database v4, as well as associated code will be available as a Zenodo deposition at https://zenodo.org/records/13236614 (DOI: 10.5281/zenodo.13236614) upon publication (44). The deposition contains domain assignments for TED, PDB files for novel folds and individual domain assignments from Chainsaw, Merizo and UniDoc to facilitate further benchmarking efforts. Individual protein annotations can also be browsed from the TED website: https://ted-dev.cathdb.info. The code for calculating consensus domain chopping, domain quality, Foldclass embedding, search, and GO term analysis is available via the PSIPRED github repository (https://github.com/psipred/ted-tools). The code for globularity prediction is part of CATH-AlphaFlow (https://github.com/UCLOrengoGroup/cath-alphaflow). The code for the TED website is available at TED-web (https://github.com/UCLOrengoGroup/ted-web).

## References

[1] Varadi M, Anyango S, Deshpande M, Nair S, Natassia C, Yordanova G, Yuan D, Stroe O, Wood G, Laydon A, Žídek A (2022). AlphaFold Protein Structure Database: massively expanding the structural coverage of protein-sequence space with high-accuracy models. Nucleic Acids Res.

[2] Varadi M, Bertoni D, Magana P, Paramval U, Pidruchna I, Radhakrishnan M, Tsenkov M, Nair S, Mirdita M, Yeo J, Kovalevskiy O (2023). AlphaFold Protein Structure Database in 2024: providing structure coverage for over 214 million protein sequences. Nucleic Acids Res.

[3] Borkakoti N, Thornton JM (2023). AlphaFold2 protein structure prediction: Implications for drug discovery. Curr Opin Struct Biol.

[4] Durairaj J, Waterhouse AM, Mets T, Brodiazhenko T, Abdullah M, Studer G, Tauriello G, Akdel M, Andreeva A, Bateman A, Tenson T (2023). Uncovering new families and folds in the natural protein universe. Nature.

[5] Barrio-Hernandez I, Yeo J, Jänes J, Mirdita M, Gilchrist CLM, Wein T, Varadi M, Velankar S, Beltrao P, Steinegger M (2023). Clustering predicted structures at the scale of the known protein universe. Nature.

[6] Bordin N, Sillitoe I, Nallapareddy V, Rauer C, Lam SD, Waman VP, Sen N, Heinzinger M, Littmann M, Kim S, Velankar S (2023). AlphaFold2 reveals commonalities and novelties in protein structure space for 21 model organisms. Commun Biol.

[7] Dustin Schaeffer R, Zhang J, Medvedev KE, Kinch LN, Cong Q, Grishin NV (2024). ECOD domain classification of 48 whole proteomes from AlphaFold Structure Database using DPAM2. PLoS Comput Biol.

[8] Sillitoe I, Bordin N, Dawson N, Waman VP, Ashford P, Scholes HM, Pang CSM, Woodridge L, Rauer C, Sen N, Abbasian M (2021). CATH: increased structural coverage of functional space. Nucleic Acids Res.

[9] Cuff AL, Sillitoe I, Lewis T, Redfern OC, Garratt R, Thornton J, Orengo CA (2009). The CATH classification revisited--architectures reviewed and new ways to characterize structural divergence in superfamilies. Nucleic Acids Res.

[10] Cheng H, Schaeffer RD, Liao Y, Kinch LN, Pei J, Shi S, Kim B-H, Grishin NV (2014). ECOD: an evolutionary classification of protein domains. PLoS Comput Biol.

[11] Bateman A, Birney E, Cerruti L, Durbin R, Etwiller L, Eddy SR, Griffiths-Jones S, Howe KL, Marshall M, Sonnhammer ELL (2002). The Pfam Protein Families Database. Nucleic Acids Res.

[12] Bateman A, Coin L, Durbin R, Finn RD, Hollich V, Griffiths-Jones S, Khanna A, Marshall M, Moxon S, Sonnhammer ELL, Studholme DJ (2004). The Pfam protein families database. Nucleic Acids Res.

[13] Lees J, Yeats C, Perkins J, Sillitoe I, Rentzsch R, Dessailly BH, Orengo C (2012). Gene3D: a domain-based resource for comparative genomics, functional annotation and protein network analysis. Nucleic Acids Res.

[14] Orengo CA, Michie AD, Jones S, Jones DT, Swindells MB, Thornton JM (1997). CATH--a hierarchic classification of protein domain structures. Structure.

[15] Lewis TE, Sillitoe I, Dawson N, Lam SD, Clarke T, Lee D, Orengo C, Lees J (2018). Gene3D: Extensive prediction of globular domains in proteins. Nucleic Acids Res.

[16] Murzin AG, Brenner SE, Hubbard T, Chothia C (1995). SCOP: a structural classification of proteins database for the investigation of sequences and structures. J Mol Biol.

[17] Fox NK, Brenner SE, Chandonia J-M (2014). SCOPe: Structural Classification of Proteins--extended, integrating SCOP and ASTRAL data and classification of new structures. Nucleic Acids Res.

[18] Hadley C, Jones DT (1999). A systematic comparison of protein structure classifications: SCOP, CATH and FSSP. Structure.

[19] Day R, Beck DAC, Armen RS, Daggett V (2003). A consensus view of fold space: Combining SCOP, CATH, and the Dali Domain Dictionary. Protein Sci.

[20] Lau AM, Kandathil SM, Jones DT (2023). Merizo: a rapid and accurate protein domain segmentation method using invariant point attention. Nat Commun.

[21] Wells J, Hawkins-Hooker A, Bordin N, Paige B, Orengo C (2023). Chainsaw: protein domain segmentation with fully convolutional neural networks. bioRxiv.

[22] Zhu K, Su H, Peng Z, Yang J (2023). A unified approach to protein domain parsing with inter-residue distance matrix. Bioinformatics.

[23] van Kempen M, Kim SS, Tumescheit C, Mirdita M, Lee J, Gilchrist CLM, Söding J, Steinegger M (2023). Fast and accurate protein structure search with Foldseek. Nat Biotechnol.

[24] Steinegger M, Söding J (2017). MMseqs2 enables sensitive protein sequence searching for the analysis of massive data sets. Nat Biotechnol.

[25] Kandathil SM, Lau AM, Buchan DWA, Jones DT (2024). Foldclass and Merizo-search: embedding-based deep learning tools for protein domain segmentation, fold recognition and comparison. biorXiv.

[26] Du D, Wang Z, James NR, Voss JE, Klimont E, Ohene-Agyei T, Venter H, Chiu W, Luisi BF (2014). Structure of the AcrAB–TolC multidrug efflux pump. Nature.

[27] Tai C-H, Paul R, Dukka KC, Shilling JD, Lee B (2014). SymD webserver: a platform for detecting internally symmetric protein structures. Nucleic Acids Res.

[28] Kajava AV, Steven AC (2006). Advances in Protein Chemistry.

[29] Mesdaghi S, Price RM, Madine J, Rigden DJ (2023). Deep Learning-based structure modelling illuminates structure and function in uncharted regions of β-solenoid fold space. J Struct Biol.

[30] Chakrabarty B, Parekh N (2022). DbStRiPs: Database of structural repeats in proteins. Protein Sci.

[31] Laitaoja M, Valjakka J, Jänis J (2013). Zinc coordination spheres in protein structures. Inorg Chem.

[32] Li T, Bonkovsky HL, Guo J-T (2011). Structural analysis of heme proteins: implications for design and prediction. BMC Struct Biol.

[33] Ekman D, Light S, Björklund ÅK, Elofsson A (2006). What properties characterize the hub proteins of the protein-protein interaction network of Saccharomyces cerevisiae?. Genome Biol.

[34] Apic G, Gough J, Teichmann SA (2001). Domain combinations in archaeal, eubacterial and eukaryotic proteomes. J Mol Biol.

[35] Batey S, Nickson AA, Clarke J (2008). Studying the folding of multidomain proteins. HFSP J.

[36] Zhou X, Hu J, Zhang C, Zhang G, Zhang Y (2019). Assembling multidomain protein structures through analogous global structural alignments. Proc Natl Acad Sci U S A.

[37] Hou Y, Xie T, He L, Tao L, Huang J (2023). Topological links in predicted protein complex structures reveal limitations of AlphaFold. Commun Biol.

[38] Jones DT, Thornton JM (2022). The impact of AlphaFold2 one year on. Nat Methods.

[39] Wayment-Steele HK, Ojoawo A, Otten R, Apitz JM, Pitsawong W, Hömberger M, Ovchinnikov S, Colwell L, Kern D (2024). Predicting multiple conformations via sequence clustering and AlphaFold2. Nature.

[40] Del Alamo D, Sala D, Mchaourab HS, Meiler J (2022). Sampling alternative conformational states of transporters and receptors with AlphaFold2. Elife.

[41] Mirdita M, Schütze K, Moriwaki Y, Heo L, Ovchinnikov S, Steinegger M (2022). ColabFold: making protein folding accessible to all. Nat Methods.

[42] Bordin N, Sillitoe I, Lees JG, Orengo C (2021). Tracing Evolution Through Protein Structures: Nature Captured in a Few Thousand Folds. Front Mol Biosci.

[43] Chothia C (1992). One thousand families for the molecular biologist.

[44] Lau A, Bordin N, Kandathil S, Sillitoe I, Waman V, Wells J, Orengo C, Jones DT (2024). The Encyclopedia of Domains (TED) structural domains assignments for AlphaFold Database v4.

[45] Zhang Y, Skolnick J (2005). TM-align: a protein structure alignment algorithm based on the TM-score. Nucleic Acids Res.

[46] Taylor WR (1999). Protein structural domain identification. Protein Eng.

[47] (2005). Conservation of Orientation and Sequence in Protein Domain–Domain Interactions. J Mol Biol.

[48] Holten D (2006). Hierarchical edge bundles: visualization of adjacency relations in hierarchical data. IEEE Trans Vis Comput Graph.

[49] Pedersen TL (2024). ggraph: An Implementation of Grammar of Graphics for Graphs and Networks.

[50] Lewis TE, Sillitoe I, Lees JG (2018). cath-resolve-hits: a new tool that resolves domain matches suspiciously quickly. Bioinformatics.

[51] Frishman D, Argos P (1995). Knowledge-based protein secondary structure assignment. Proteins.

[52] Zhou AN, Jiang Y, Bergquist TR, Lee AJ, Kacsoh BZ, Crocker AW, Lewis KA, Georghiou G, Nguyen HN, Hamid MN, Davis L (2019). The CAFA challenge reports improved protein function prediction and new functional annotations for hundreds of genes through experimental screens. Genome Biol.

[53] UniProt Consortium (2023). UniProt: the Universal Protein Knowledgebase in 2023. Nucleic Acids Res.

[54] Orengo CA, Taylor WR (1996). SSAP: sequential structure alignment program for protein structure comparison. Methods Enzymol.

[55] Laskowski RA, Jabłońska J, Pravda L, Vařeková RS, Thornton JM (2018). PDBsum: Structural summaries of PDB entries. Protein Sci.

[56] Valdar WSJ (2002). Scoring residue conservation. Proteins.

[57] Kabsch W, Sander C (1983). Dictionary of protein secondary structure: pattern recognition of hydrogen-bonded and geometrical features. Biopolymers.

[58] Biswas T, Houghton JL, Garneau-Tsodikova S, Tsodikov OV (2012). The structural basis for substrate versatility of chloramphenicol acetyltransferase CATI. Protein Sci.

