# Supplementary material for: Exploring structural diversity across the protein universe with The Encyclopedia of Domains
Source: Science. Author manuscript; Available in PMC 2026 Mar 14. (PMC7618865; doi:10.1126/science.adq4946)
Supplement: Supplementary [file EMS212919-supplement-Supplementary.pdf]

Supplementary Materials

Supplementary Methods

Figs. S1 to S19

Tables S1 to S4

Supplementary Methods

Datasets

Our analysis was carried out on Version 4 of the AFDB, containing models for 214,683,829 UniProt sequences. To avoid overrepresentation bias, most of our initial analysis was carried out on a non-redundant subset of 188,914,411 sequence unique AFDB model (TED-100). There are 38,944,835 targets which are sequence-redundant - 13,175,417 are in TED-100 (sequence representatives were selected as the first target in a sorted list), and the remaining 25,769,418 are unique to the TED-redundant dataset.

Deriving a unified assignment of domains in the AFDB

Both TED-100 and TED-redundant datasets were subjected to a consensus domain parsing workflow which made use of three segmentation methods: Merizo, Chainsaw and UniDoc, before deriving a consensus between their outputs. Each method is provided with the structure of the AFDB model and returns the predicted domain ranges. In the case of UniDoc, which does not classify NDRs, models were first parsed using Merizo to remove NDRs before subsequent segmentation into domains with UniDoc. This two-step procedure for generating UniDoc domains where NDRs are first removed, is necessary as otherwise the presence of NDRs can mislead UniDoc into classifying full chains as single domains. Furthermore, removal of NDRs via using only plDDT scores is insufficient, as previously shown (*20*). Overall, 400,444,974, 328,956,414 and 366,117,430 putative domain regions were identified by Merizo, Chainsaw and UniDoc respectively (Table S1 and Fig. S2), requiring several months of computation using the CS Cluster at UCL. Compute hardware consisted of a mixture of both CPUs (approximately 400 cores at peak) and GPUs (up to approximately 100 NVIDIA GTX 1080 Ti or RTX 2080 Ti). GPUs were utilised for deep-learning based methods Merizo and Chainsaw, while UniDoc was run using only CPUs. Further details on how consensus domains were derived are described in the Supp. Methods.

Domain classification using Foldseek

Foldseek (*23*) (version a435618b95ba95cbfe74dcbc8b4bd2720547d285) easy-search was benchmarked to assess tentative homology and fold assignments thresholds on a curated set of CATH domains. We created a dataset of 3,186 domains representative of CATH classes 1 to 4 clustered at 30% sequence identity with equivalent superfamily and boundary assignments in agreement with SCOP. Using a genetic algorithm, we identified thresholds for superfamily (H-level) and fold (T-level) level matches in CATH at a 98% precision level with the following parameters: E-value cutoff of 0.108662, minimum coverage of 0.366757, coverage mode 5 (shortest sequence is at least 36.6757% of the longest sequence) and sensitivity 10. Further post processing on the raw results from Foldseek was applied by using custom thresholds for H-level and T-level hits. At this precision level, the recall at H and T levels is approximately 0.59 and 0.71.

We selected as valid H-level hits any results that passed the thresholds set to Foldseek, with an additional cut-off based on a TM-score over 0.56, a coverage threshold of 0.367 and an E-value below 0.019. Hits at the fold level (T) required different cutoffs, with results considered as valid hits if passing the Foldseek E-value cutoff, a coverage cutoff of 0.786 and a minimum TM-score of 0.42. Using these thresholds, we scanned all 324,389,697 domains with a medium/high confidence against a library of 31,574 CATH SSG5 (Structural Similarity Groups, 5Å, where all CATH superfamily members are within 5Å when superposed) (*9*), resulting in 193,939,494 hits at the H-level and 16,026,530 hits at the fold level, leaving 114,423,673 domains with no labels assigned at this stage.

Domain classification using embedding similarity

To further reduce the number of unmatched domains, we subjected the remaining domains not matched by Foldseek to an in-house structure embedding search method (Merizo-search) in order to identify further matches to our CATH SSG5 representative dataset. Merizo-search uses a deep learning method called Foldclass that makes use of an equivariant graph neural network to encode a domain structure into a fixed-size embedding (*25*). A query domain is embedded using Foldclass and then compared against a database of CATH domain embeddings, with similarity determined via cosine distance between the target and CATH domain embeddings. To further verify the top match, TM-align (*45*) is employed to align the query coordinates against the CATH domain corresponding to the closest domain embedding. The nearest neighbour CATH domain is considered a hit if the TED domain achieves a TM-align score greater than 0.5 (normalised by the length of the TED domain).

Sequence clustering of TED-100 domains

The sequences of TED-100 domains were clustered using MMseqs2 (version 22a77e22a77eeb1b6c64f1c5f04e1480b4705bb3fbc897), enforcing a minimum sequence identity of 50% and at least 90% coverage of the shorter sequence against the longer sequence (coverage mode 5). Sequence clusters were then populated using CATH labels identified using Foldseek and Foldclass (*25*) methods (see sections 'Domain classification using Foldseek' and 'Domain classification using embedding similarity'). A full breakdown of the number of labelled and unlabelled sequence clusters, and the number of domains comprising these clusters is available as Table S1.

Novel Domain Identification Workflow

As a starting point for finding domains with putative novel folds, we filtered the remaining CATH-unlabelled clusters using three criteria to remove problematic structures and those predicted with low confidence. These filters included: two metrics that describe the globularity of the structure (radius of gyration and packing density described in Bordin et al. (*6*); Fig. S19), the number of secondary structure elements, as well as a 'plDDT_{80}' metric (Fig. S20) which we make use of in our methodology. Full details on how these filters were applied can be found in the Supp. Methods.

The filtered sequence cluster representatives are then subjected to layers of further clustering and filtering (Foldseek commands are described in the Supp. Methods). The next step of the workflow involves iterative searches of the remaining cluster representatives against the PDB and CATH (*8*), ECOD (*10*) and SCOPe (*17*) domain libraries using Foldseek (*23*). These searches use Foldseek's easy-search using (exhaustive) TM-align mode, eliminating matches with a TM-align score greater than 0.56 and coverage of 60%. The reason for using a slightly unorthodox TM-align threshold of 0.56 is to compensate for Foldseek approximating the true TM-align score (*23*), meaning that occasionally, false-positive matches are found when using the standard threshold of 0.5.

Identifying putative novel folds

Although we consider the remaining 240,674 candidate clusters as our main working set of possible novel domain folds, further processing was carried out to try to identify a core subset of domains which were both novel and consistent with the features of well-folded protein domains. Firstly, we separated out the subset of clusters composed of high-symmetry domains. These domains may be composed of known repetitive units, for example, WD40 repeats in beta propellers, but arranged in a manner which confers novelty. High internal symmetry domains were identified using the Z-score returned by the SymD program (*27*). We used a threshold of Z-score > 9.0 to indicate high-symmetry domains, following the original SymD method paper which used cutoff values of 8 and 10 (Fig. S10).

Detecting properly chopped novel fold domains

The remaining low-symmetry clusters were assessed on domain chopping quality using a retrained Foldclass network. This new network is trained as a simple binary classifier to distinguish between existing real CATH S30 domains (positives) and random crops of the associated full length protein chains (negatives). For every true domain, either the true domain is taken with a positive label or a random crop is sampled from the same chain with a negative label. A probability of 0.5 was used for this to ensure a balanced training set. The loss function for training is binary cross entropy, all other training details are the same as for FoldClass. The trained network thus outputs a score between 0 to 1 which indicates the likelihood of a given set of coordinates having been chopped in a manner consistent with the existing CATH domain database. We use a moderate threshold of quality score < 0.5 to eliminate any domains which are less likely than not to be correctly chopped, but without rejecting too many clusters that might contain reasonable candidates for globular domains with potentially novel folds. As an additional filter, we also made use of another older domain parsing algorithm (*46*) which is generally very good at identifying single domain proteins based on compactness (domains that were not identified as being single domain with 100% confidence were removed).

The remaining domains were then subjected to increasingly sensitive Foldseek comparisons with the current CATH, ECOD and SCOPe domain databases (*8*, *10*, *17*), with commonly-used thresholds of TM-score threshold of 0.5 and query domain coverage of 60%. The TM-score was taken as *max(qtmscore, ttmscore)* so that both the TM-scores normalised by the candidate domain length and normalised by the library domain would be considered. The final stage was

to use exhaustive comparisons against the domain libraries using TM-align directly (*45*). TM-align is generally more sensitive than Foldseek even in its exhaustive mode, and indeed we found further matches (TM-score > 0.5 and query coverage > 60%) to reduce the final list from 24,653 to 7,427 potential novel domain folds. This set of domains represents the set we feel most confident about defining as well-folded domains with no significant structural similarity to current PDB chains (as of December 2023) or current structural domain libraries (as of February 2024).

Using the Foldclass embeddings we ranked the final list of domains in order of novelty by calculating the mean Euclidean distance between the embedding vector of each candidate domain and the *k*-nearest neighbours ($k=50$) in CATH, ECOD and SCOPe.

GO term analysis of novel and repeat domains

To try to evaluate the possible functions of our final repeat and novel fold domains, we used a purely sequence-based predictor (GOfocus) which makes use of a deep dilated convolutional network to predict a set of slimmed GO terms. Further details of the model can be found in the Supp. Methods.

Evaluating domain interactions

To identify which pairs of domain superfamilies interact, and the degree of conservation in the interaction geometry for that pair of superfamilies, domains were defined as interacting if there were at least 3 Cβ atom pairs (Cα in the case of Gly residues) within 8Å between the two domains. An ISP is the collection of all instances of such contacting pairs of domains belonging to specific superfamilies, and an ISP is uniquely identified by the CATH H-level (homologous superfamily) labels comprising it (e.g. 1.10.10.10 - 3.20.20.10). ISPs are identified independently of the order of occurrence of the component domains in the chain sequence and are only defined for domain pairs within the same chain. For TED domains, we filter each putative set of interacting domains using the PAE data (*2*) supplied with each AFDB chain prediction.

Conservation in interaction patterns was assessed using the Conservation of Interaction Orientation (CIO) measure of Littler and Hubbard (*47*). A CIO score of 0 indicates complete conservation in the interaction geometry (i.e. interaction vectors for a given ISP are absolutely coincident), and a score of 1 indicates a uniformly random angular distribution of vectors. Interaction vectors were only computed for interacting domain pairs as defined above, and all instances of a given ISP are first placed in a common frame of reference by aligning on a common reference structure for one of the domains in the pair, using TM-align (*45*). We also assessed the number of occurrences of ISPs common to both TED and CATH 4.3, expressing relative enrichment as the $\log_2$(fold change) of pairs in TED relative to CATH.

To visualise the sets of ISPs in CATH and TED, we used hierarchical edge bundling plots (*48*) as implemented in the 'ggraph' R package (*49*), using the CATH hierarchy with a virtual common root for classes 1-4 as the guide dendrogram (Fig. S13). To identify hub domains, we

first take each superfamily and determine the ISPs it is involved in within CATH and TED. A superfamily involved in k ISPs is classified as a 'small', 'medium' or 'large' hub when $k<4$, $4<=k<=7$, and $k>7$, respectively, following the thresholds defined in Ekman et al. (*33*) but applied to domains rather than full-length proteins.

Sequences not modelled in AFDB

The AFDB excludes certain sequences from the UniProt 2021_04 release (https://alphafold.ebi.ac.uk/faq), and consequently these are not covered by TED. To get an idea of the makeup of these sequences, we obtained UniProt release 2021_04 and the 'accessions_ids.csv' file for AFDB v4 from https://ftp.ebi.ac.uk/pub/databases/alphafold/. Using the accessions in the two data sets, and limiting ourselves to sequences at least 16 residues long (as these are unlikely to contain nontrivial domains), we find that a total of 10,749,213 sequences do not appear in the AFDB. Using taxonomic data in UniProt we find that of these, 5,304,757 are listed as being from viruses. 554,700 and 3,360,117 of these sequences are longer than 2700 and 1280 residues, respectively (up to 2700 residues are allowed for SwissProt or reference proteome sequences, whereas 1280 is the upper limit for other sequences). Although we are unable to provide consensus domain counts for the set of 10.8 million unmodelled sequences (as they do not have structures in AFDB), we can estimate domain counts based on the length distribution of these targets. Under the assumption that they exhibit a similar domain count-to-length ratio as the current AFDB sequences, and using TED100 to estimate the number of domains per residue in all modelled UniProt entries, we estimate that there may be roughly 50 million domains not yet covered by the AFDB, with the caveat that the distribution of non-domain residues could be different for longer sequences.

Validation of homology assignments using Hidden Markov Models

Consensus domain sequences with an H-level or T-level assignment by Foldseek were scanned against Hidden Markov Models built from 62,915 CATH representatives clustered at 95% sequence identity. The resulting output from HMMsearch with an E-value cut-off set at 1e-3 were subsequently post-processed using cath-resolve-hits (*50*) using a minimum coverage of 80% and minimum bitscore set at 25. Resulting superfamily code assignments for H hits were checked for full matches (identical CATH codes predicted) or fold matches (first three of the CATH digits identical, different fourth level assignment between the two methods). For T-level assignments by Foldseek we compared only the first three digits in the CATH code assigned by the HMMs, returning a valid match only if the first three digits are identical, returning a non-match otherwise.

Evaluation of domain coverage via sequence and structural searches

Full, non-redundant sequences for AFDB were scanned against Hidden Markov Models built from 62,915 CATH representatives clustered at 95% sequence identity. The resulting output from HMMsearch with an E-value cut-off set at 1e-3 were subsequently post-processed using

cath-resolve-hits (*50*) using a minimum coverage of 80% and minimum bitscore set at 25. The tool returns the best non-overlapping set of domain assignments on each AFDB sequence.

Deriving a unified assignment of domains in the AFDB

The predicted domains on full length AFDB targets by Merizo, Chainsaw and UniDoc were filtered to remove any segments that are fewer than 5 residues, and any domains which are fewer than 25 residues. To determine a common set of putative domains identified by all three methods, domains are classified into high, medium or low consensus categories depending on the level of agreement between the predictions of the three methods (Fig. S14). Specifically, given a set of N domain predictions for a given target, we construct an N-by-N matrix of pairwise sequence overlap scores between each domain, thresholded at 70% coverage to generate an adjacency matrix. This matrix can be treated as a network graph, with the size of each internal graph component indicative of the confidence level (3, 2 and 1 indicating high, medium and low confidence, respectively).

For TED-100, of which there are 188 million unique sequences, the consensus is derived for each individual protein. High, medium and low confidence categories reflect the agreement of predictions by either three, two or single methods. The final domain ranges reported, are taken as the intersection between the regions in agreement with each other. For domain counts and subsequent downstream analysis tasks, only the high and medium categories are considered due to their non-overlapping nature. Due to the nature of their derivation, high and medium consensus domains cannot overlap.

For TED-redundant, which encompasses 38,944,835 sequence-redundant targets, we calculated the consensus domains for the 25,769,418 targets not in TED-100 using the same approach (calculated per chain). Overall, we identified 324,482,131 domains in TED-100 (195,229,271 at high consensus and 129,160,426 at medium consensus) and 40,417,498 domains in TED-redundant (24,483,532 at high consensus and 15,933,966 at medium consensus). The total domain count for TED is taken as the sum of TED-100 and TED-redundant and is 364,807,195 domains, covering all 214,683,829 AFDB targets.

As an alternative view of TED-redundant, we additionally derived a second consensus that takes into all models for each redundant sequence. This covers all 38,944,835 sequence-redundant targets (including the 13,175,417 representatives part of TED-100). This consensus uses dynamic thresholding for high and medium consensus categories due to the variable number of models being considered for each sequence. Thresholds were derived as *threshold* = $3Mk$, where M is the number of targets (2 minimum), and k is either 0.66 or 0.33 for high and medium confidence levels, respectively. For the TED-redundant-multi set only, high consensus domains are given priority over those of medium consensus, with any in the latter category overlapping with high consensus domains discarded.

Novel Domain Identification Workflow

Our novel domain identification workflow makes use of several filters that were applied to non-CATH-labelled cluster representatives. First, the representatives of the 41,879,858 domain clusters were assessed using the normalised radius of gyration and packing density described in Bordin et al. (*6*). Globular TED domains were determined with CATH-AlphaFlow as those satisfying both criteria of having a normalised radius of gyration of below 0.356, and a packing density of greater than 10.333 (Fig. S19). Cutoff values were determined as the 5th percentile of both metrics calculated from the 193,939,492 TED-100 domains with H-level CATH assignments, as these domains were of sufficient quality to be representative of domains in the AFDB.

For secondary structure elements (SSE) filtering, domains were excluded from consideration as novel domains if they contained fewer than 6 SSEs (helices and strands), as determined by STRIDE (*45*, *51*). STRIDE counts even very short regions (2 residues) as separate secondary structures, so a threshold of 6 elements in total was deemed a sensible threshold to allow us to identify novel domain architectures. Applying both the globularity and SSE filters to the 41,879,858 unlabelled clusters, results in 13,820,550 passing both criteria.

Finally, we apply a plDDT filter on the remaining domains, which we term the '$plDDT_{80}$' metric. The $plDDT_{80}$ is calculated as the average plDDT of the top 80% of residues ranked by plDDT. We found $plDDT_{80}$ preferable to the simple average as it prevented removing clusters that are mainly well-folded but have unfolded tail regions. Applying a $plDDT_{80}$ >= 90 filter on the remaining 13,820,550 clusters yields a final set of 8,612,318 clusters which pass all three (globularity, SSE and $plDDT_{80}$) criteria, encompassing 19,816,697 domains within them.

GO term analysis of novel and repeat domains

To try to evaluate the possible functions of our final repeat and novel fold domains, we used a purely sequence-based predictor called GOfocus, which performed well in the 4th CAFA experiment (*52*) [https://biofunctionprediction.org/cafa/]. The model comprises a 1-D dilated convolutional network with 4 blocks of 4 dilated convolutional layers per block, with dilations cycling through 1, 2, 4 and 8, a filter size of 3, 512 channels and residual connections across each layer. After the dilated convolutional blocks, adaptive max-pooling is used to reduce the (L,512) tensor to fixed dimensions (16,512) which are then flattened and used as input features for a two layer fully connected network with ReLU non-linearity and dropout rate set to 0.1. Sixteen separate networks were trained with different non-overlapping subsets of a slimmed set of 8360 GO term labels. Subsets were chosen to keep terms with similar observed frequencies in the sequence data bank grouped together. Training was carried out on the UniRef50 subset of sequences in the December 2019 release of SwissProt and TrEMBL and their respective (non-IEA) GO terms (*53*). Focal loss was used as the loss function and training was carried out with the standard Adam optimizer with a learning rate of 1e-4. Testing this model on the CAFA3 benchmark test data produced an $F1_{max}$ score of 0.65 for Molecular Function terms.

MMseqs commands for sequence clustering

Number of sequences input to clustering: 324,389,698
Number of clusters generated: 120,748,700

Clusters generated using:

```
mmseqs easy-linclust domain_sequences.fasta results tmp --cov-mode 5 -c 0.9 --min-seq-id 0.5
```

Foldseek commands used for novel fold identification workflow

Number of models input to clustering: 8,612,318
Number of clusters generated: 1,036,525

Clusters generated using:

```
foldseek cluster foldseek.db result tmp -s 4 -e 0.001 -c 0.7 --cov-mode 5 --tmscore-threshold 0.56 --min-seq-id 0.2 --seq-id-mode 1 --cluster-mode 1
```

1,036,525 clusters were further clustered using below command to generate 625,802 clusters:

```
foldseek easy-cluster models result tmp -c 0.8 --tmscore-threshold 0.8 --cluster-mode 1
```

625,802 cluster representatives searched against PDB chains using:

```
foldseek easy-search models PDB result.m8 tmp -c 0.6 --cov-mode 2 --tmscore-threshold 0.5 --alignment-type 1 --format-output query,target,qlen,tlen,qtmscore,cigar
```

Output file filtered using `qtmscore > 0.56` and cigar-computed `qcov > 0.6` to generate resultant 358,491 clusters. Clusters are searched against domain libraries using:

```
foldseek easy-search models domain_db result.m8 /ssd1/jones/tmp -c 0.6 --tmscore-threshold 0.3 --alignment-type 1 --exhaustive-search 1 --format-output query,target,qlen,tlen,qtmscore,cigar
```

Results are filtered using `qtmscore > 0.56` and cigar-computed `qcov > 0.6` and cigar-computed `tcov > 0.6` to generate 240,674 clusters.

Low symmetry domains comprising 138,120 clusters (after domain quality and high-symmetry

filtering) are clustered together with domain libraries:

```
foldseek easy-cluster models_with_domain_db result tmp --tmscore-threshold 0.5 -
c 0.6 --alignment-type 1
```

This generates 39,037 clusters which are not composed of any members from the domain databases.

39,037 cluster representatives are searched against domain libraries using:

```
foldseek easy-search models domain_db result.m8 tmp -c 0.6 --cov-mode 2 --tmscore-
threshold 0.3 --alignment-type 1 --exhaustive-search 1 --format-output
query,target,qlen,tlen,qtmscore,ttmscore,rmsd,cigar
```

Results are filtered for `max(qtmscore,ttmscore) > 0.5` or `RMSD < 3` and cigar-computed `qcov > 0.6`, retaining 24,653 clusters.

Final clusters are searched again against domain libraries using manual TM-align runs and filtered using `qcov > 0.6` and `max(qtmscore,ttmscore) > 0.5` to leave 7,427 final clusters.

Detection of poor quality domain choppings using an EGNN network

Although the use of a consensus domain segmentation method produces generally good quality domains, inevitably, amongst the long tail of possible novel folds we will find candidate domains that represent either over- or under-chopped chain segments. By under-chopped we mean structures that still appear to include domain linker regions, and by over-chopped we mean structures that are similar to existing domains but which are incomplete e.g. a segment of a TIM-barrel. As such, we assessed each remaining low-symmetry cluster on chopping quality, by retraining a variant of the Foldclass network to identify tenuous domain choppings.

We employed a version of the Foldclass network (with 3 EGNN layers rather than 2) to score domains in terms of the quality of the segmentation with a two class (binary) output layer. For every domain in the 30% non-redundant CATH 4.3 domain set, we trained the network to recognize these domains as being in the positive class i.e. are correctly segmented. For the negative labelled cases, we generated an entirely random crop of the related full-length protein chain. A random 50-50 mix of positive and negative labelled cases was considered during each epoch of training.

Structural characterisation of CoA-dependent acyltransferases in TED

CATH domains for the Chloramphenicol Acetyltransferase superfamily (3.30.559.10, n=254) were scanned in an all-vs-all fashion using SSAP (*54*) and clustered with complete-linkage using cath-cluster (https://cath-tools.readthedocs.io/en/latest/tools/cath-cluster/) into 14 SSG5

(Structural Similarity Groups, 5Å), which were inspected for oligomerization state and ligand data available in the Protein Data Bank. TED domain structures with an associated H-level assignment to the 3.30.559.10 superfamily were retrieved (n=228,867) and scanned against the library of 14 SSG5s using Foldseek-TMalign with the following command:

```
foldseek easy-search pdb/ 3.30.559.10_ted_domains_models.tar.gz ssg5_vs_ted_domains_tmalign_over_0.7 tmp --max-seqs 300000 --cov-mode 5 -c 0.6 --tmscore-threshold 0.7 --alignment-type 1 --tmalign-hit-order 1 --format-output query,target,qstart,qend,qlen,qcov,tstart,tend,tlen,tcov,alnlen,pident,qtmscore,ttmscore,rmsd
```

TED hits retrieved from Foldseek were annotated with a comprehensive set of pathogens-associated TaxonIDs (n=39,664) from BV-BSRC v3.35.5 (https://www.bv-brc.org/), ENA Pathogens Portal (https://www.pathogensportal.org/) and VEuPathDB (https://veupathdb.org/veupathdb/app/). Hits were subsequently sorted by sequence similarity, TMscore, pathogenicity and plDDT, with priority for analysis given to SSGs with known ligand information in PDB, low sequence similarity to any known PDB structures, high TMscore for both query and target, and high plDDT.

For CATH experimental domains, known substrate binding residues were retrieved from the literature and PDBSum (*55*). For TED domains, predicted conserved residues were obtained by scanning the TED domain against AlphaFold/UniProt50 v4 using Foldseek server (https://search.foldseek.com/search), retrieving structural relatives with TM-Score greater than 0.70 and where conserved sites were detected by the Scorecons algorithm (*56*) on the multiple sequence alignment derived from the relatives.

The trimer for the TED domain in *Clostridium botulinum* was modelled with AlphaFold-Multimer using ColabFold 1.5.2 (*41*) with 20 recycles and amber relaxation using the following command:

```
colabfold_batch --templates --amber --num-models 5 --num-relax 5
```

Supplementary Figures

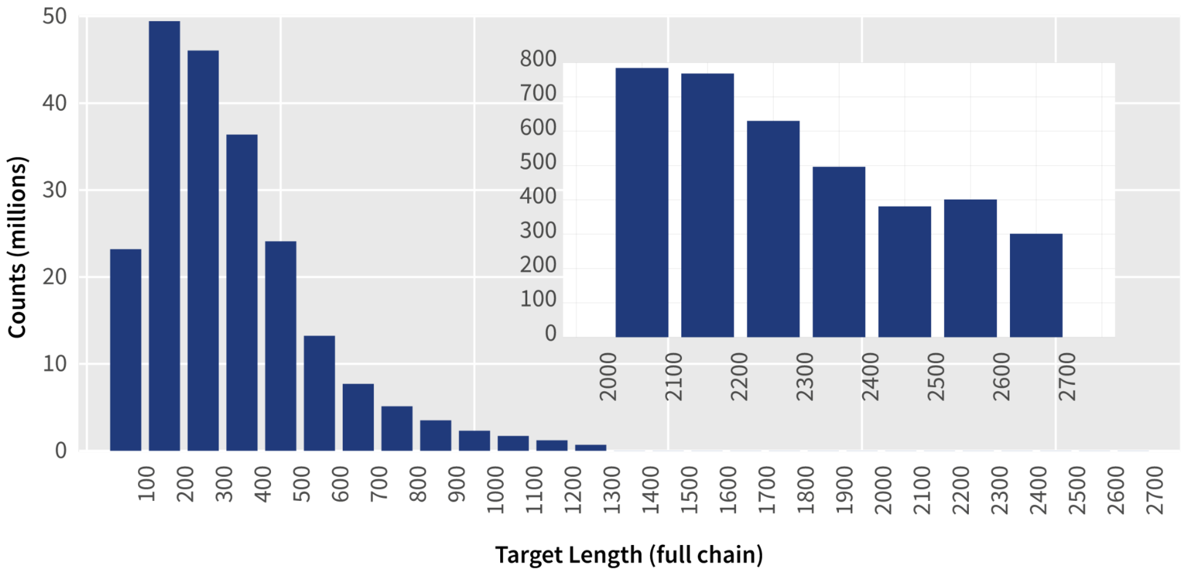

Fig. S1. Distribution of target lengths in the AFDB. Histogram encompasses the full database of full length targets in the AFDB (n=214,683,829). Inset shows the distribution of target lengths for targets longer than 2000 residues. The AFDB contains models up to a maximum length of 2700 residues (as part of the full distribution containing 214m targets).

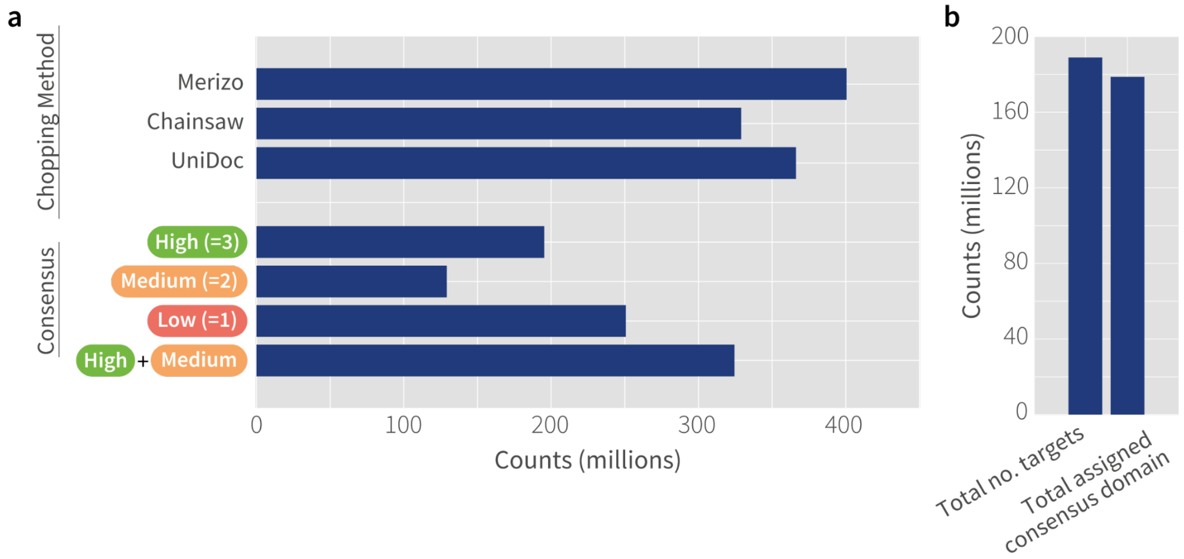

Fig. S2. Domain counts identified from consensus chopping methods in TED-100. (a) Number of domains identified from Merizo, Chainsaw and UniDoc methods. Domain regions were aggregated into three consensus levels: high (agreement between all three methods), medium (agreement between two methods) and low (regions without agreement between at least two methods). Medium and high consensus domains were taken forward for analysis. (b) Number of targets in TED-100 with consensus domain (high and medium levels) coverage. 98% of targets are covered by TED-100 in total.

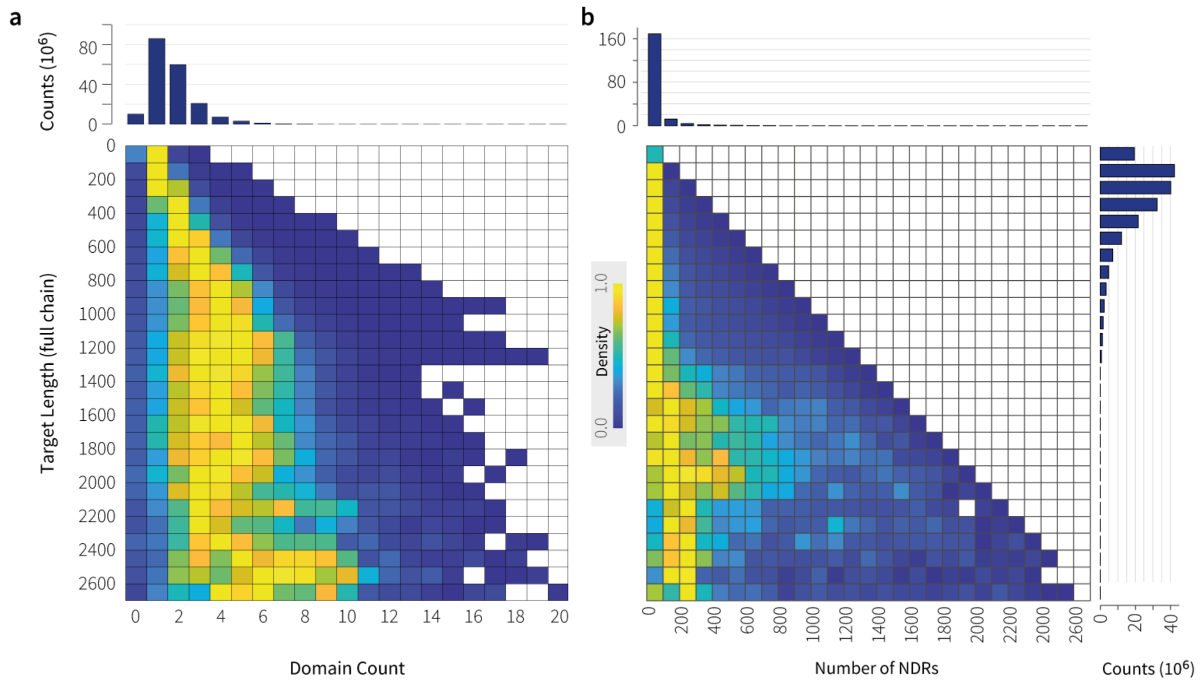

Fig. S3. Distribution of domain counts and number of NDRs across targets in TED-100. Data encapsulates 188m full length AFDB targets in TED-100. (**a**) Domain counts per target were taken as the total number of medium and high consensus domains. (**b**) NDRs were determined as residues not assigned into any domains by either of the three chopping methods (residues not in any low, medium or high consensus categories). Colour scheme has been normalised across rows. Each row represents a bin of 100 residues, up to the maximum length of 2700 for AFDB models.

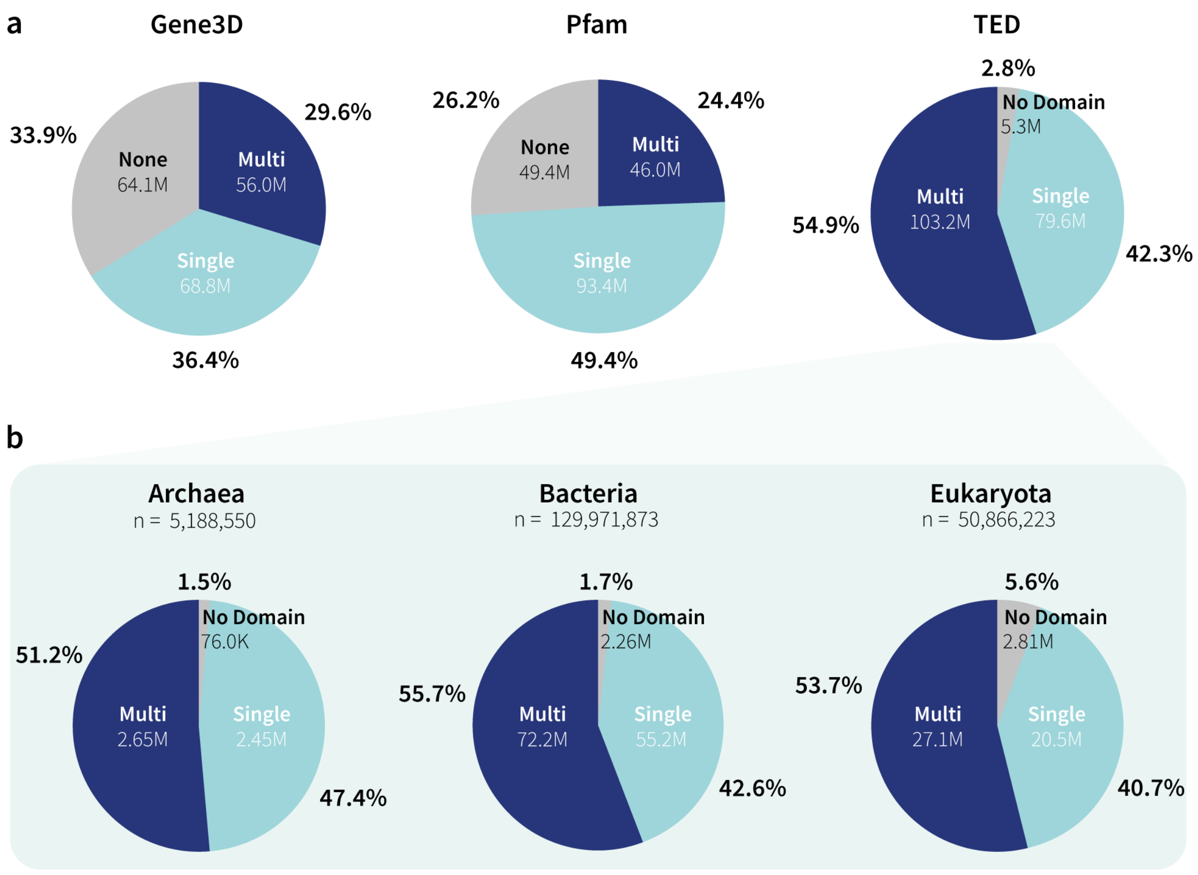

Fig. S4. Proportion of single- and multi-domain targets identified in TED-100. (**a**) Comparison of domain compositions according to Gene3D, Pfam and TED assignments. (**b**) TED-100 assignments subdivided by the three major superkingdoms.

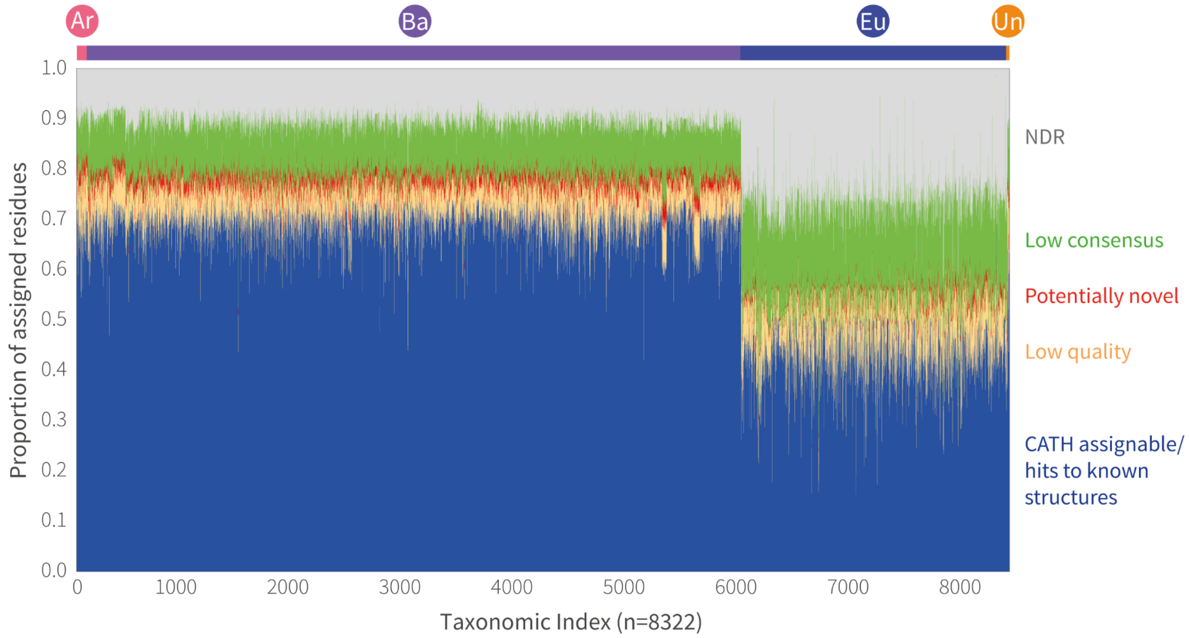

Fig. S5. Proportion of assigned residues in TED-100. Data shown represents a subset of taxa which are composed of at least 5000 targets across TED-100 (n=8322). Taxa are grouped based on UniProt superkingdoms (archaea (Ar), bacteria (Ba), eukarya (Eu) and unclassified (Un)). "CATH assignable" (blue) includes domains from sequence clusters containing at least one CATH-assigned member, as well as domains that can be matched to PDB and domain databases. "Low quality" (yellow) encapsulates domains removed due to low-globularity, few-SSE or low-plDDT filters. "Novel" (red) category includes domains considered as novel domains. "Low consensus" (green) category includes residues where medium/high consensus could not be reached, but are non-NDR. "NDR" (grey) represents residues not considered to be part of domains by any of the three domain segmentation methods.

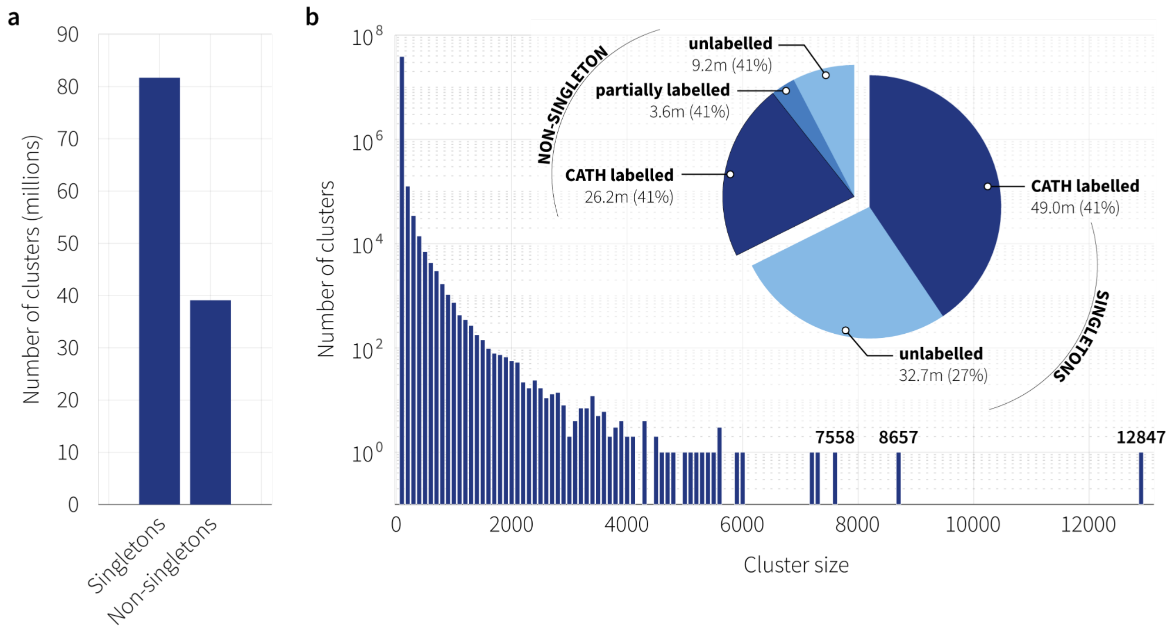

Fig. S6. Summary of sequence clusters in TED-100. (**a**) Number of singleton and non-singleton domain sequence clusters identified at 50% sequence identity and minimum overlap of 90%. (**b**) Histogram of cluster sizes for non-singleton clusters (bin width of 100). The majority of clusters are encapsulated within the first bin (below 100 members). The number of sequence members in the largest 3 bins are displayed. Inset shows the proportion of singleton and non-singleton clusters that can be assigned CATH labels. 'CATH labelled' clusters represent clusters where every member is assigned a CATH label at the superfamily or topology levels. 'Partially labelled' clusters contain at least one member with a CATH superfamily or topology level. 'Unlabelled' clusters are entirely unlabelled.

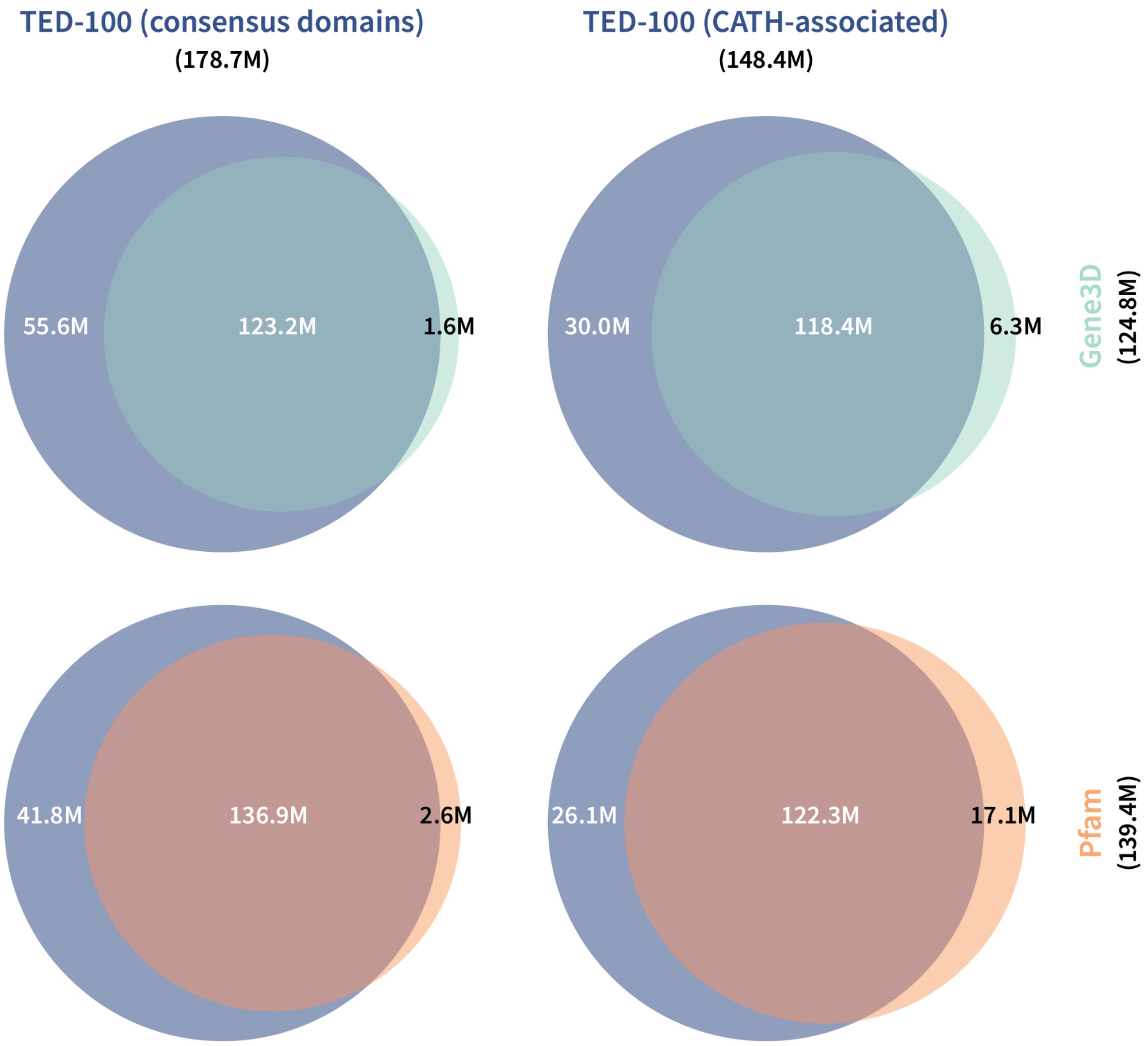

Fig. S7. Comparing the coverage of TED against Pfam and Gene3d. Venn diagrams compare the protein-level coverage of TED-100, across all proteins encompassing high and medium consensus domains (178.7 million proteins), and those that are associated with CATH-labelled clusters (148.4 million proteins), with those of Gene3D (124.8 million proteins) and Pfam (139.4 million proteins).

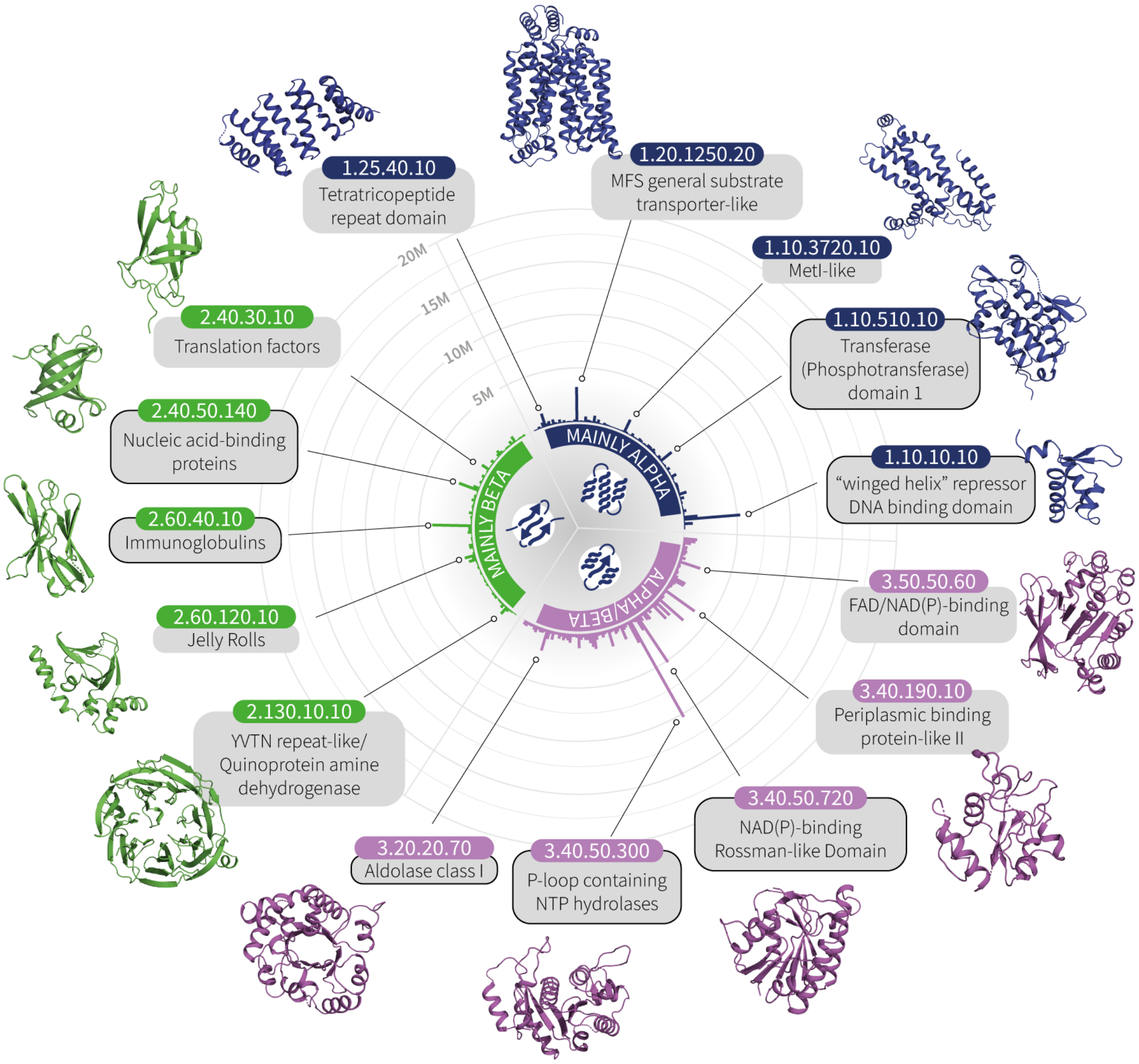

Fig. S8. Most abundant superfamilies identified in TED-100. The top 5 superfamilies per CATH class are shown, along with representative domain structures from CATH. Superfamilies that are part of the top 5 most abundant in CATH are outlined in black (Fig. S9).

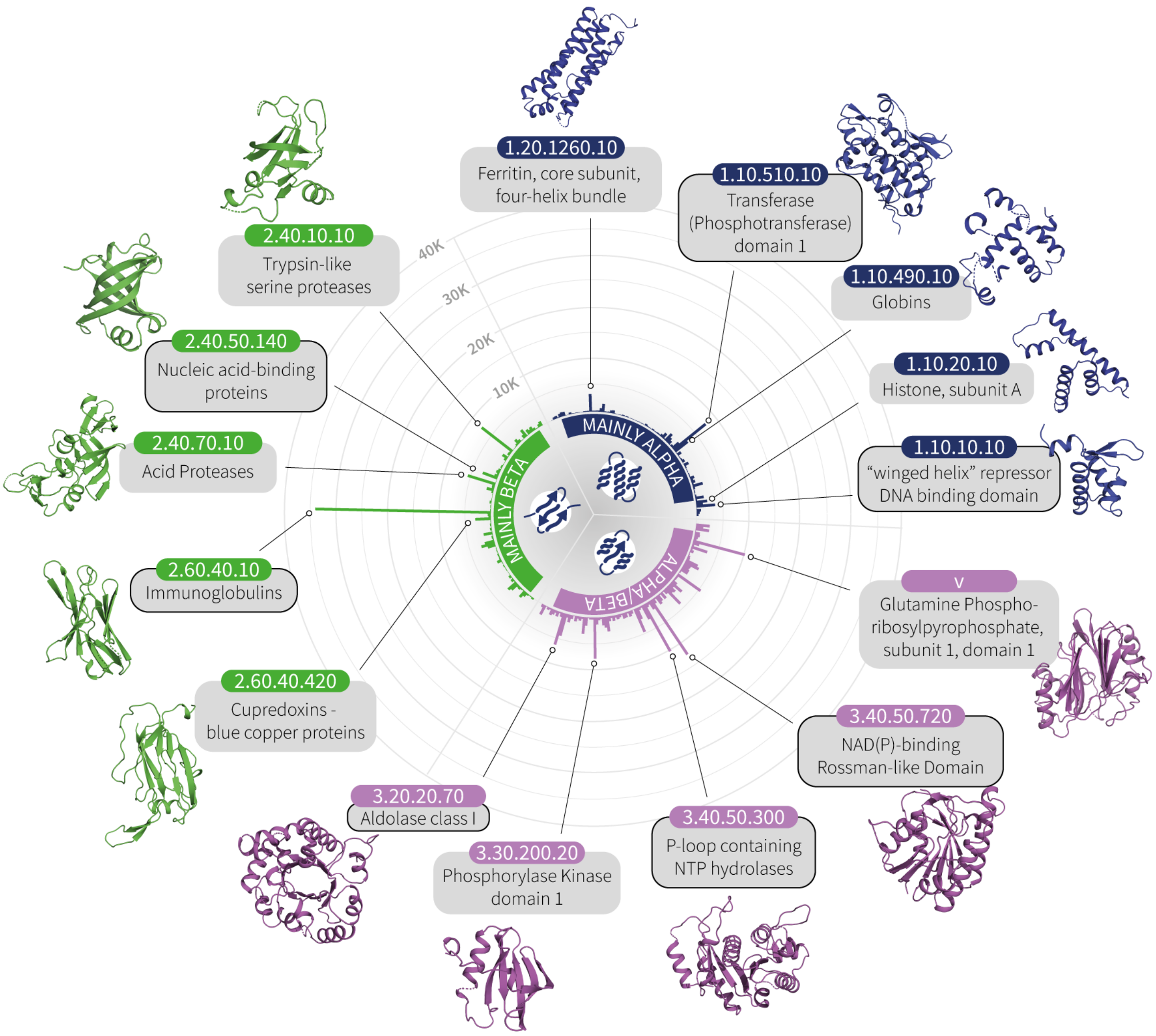

Fig. S9. Most abundant superfamilies in CATH. The top 5 superfamilies per CATH class are shown, along with representative domain structures from CATH. Superfamilies that are part of the top 5 most abundant in TED-100 are outlined in black (Fig. S8).

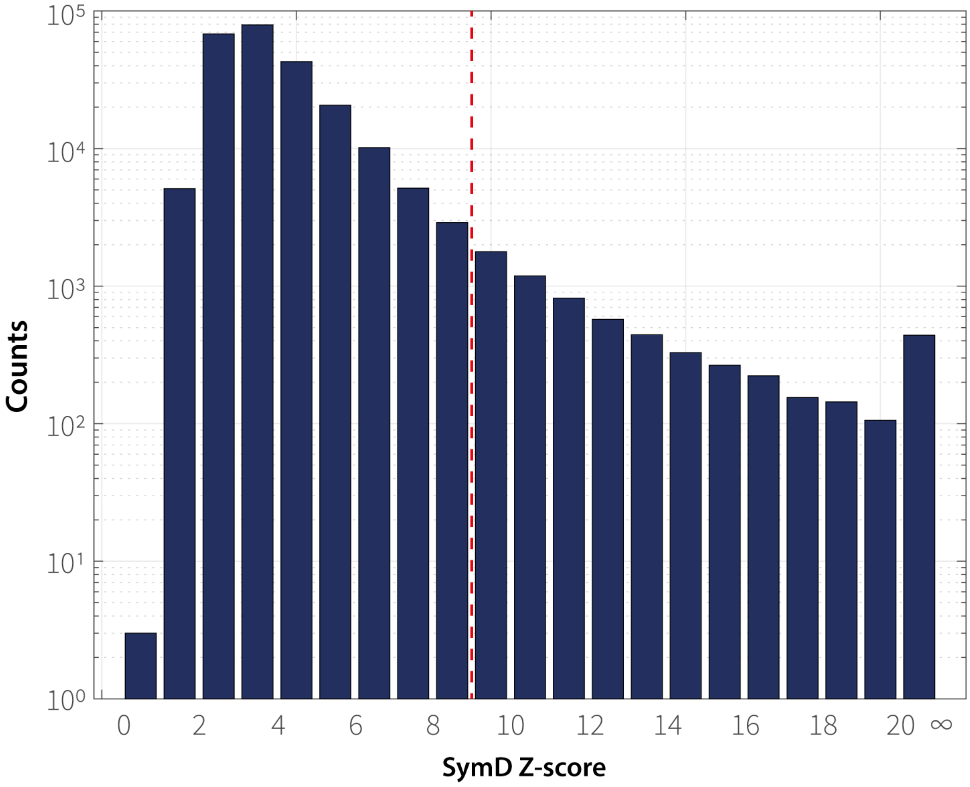

Fig. S10. Distribution of symmetry Z-scores calculated on candidate novel domains. Z-scores are calculated using the SymD program on TED domain structures directly (n=240,674). The red line demarcates the cutoff of 9 used for filtering high-symmetry domains.

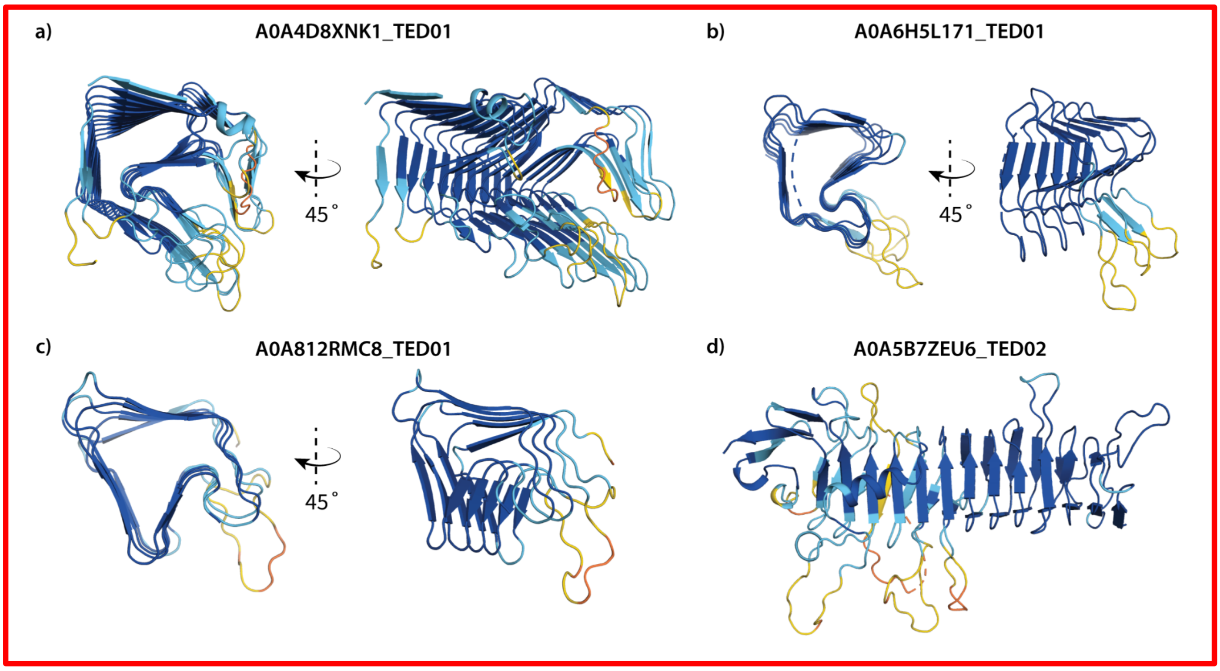

Fig. S11. Examples of extruded repeats with highly varied loop decorations. Each panel shows an example of an extruded repeat domain. Panels a-c show the projection along the extrusion axis, along with a 45˚ rotated view showing unstructured loops. Structures are coloured according to AFDB plDDT bins (dark blue/very high: plDDT >= 90, blue/high: 90 > plDDT >= 70, yellow/low: 70 > plDDT >= 50 and orange/very low: plDDT < 50).

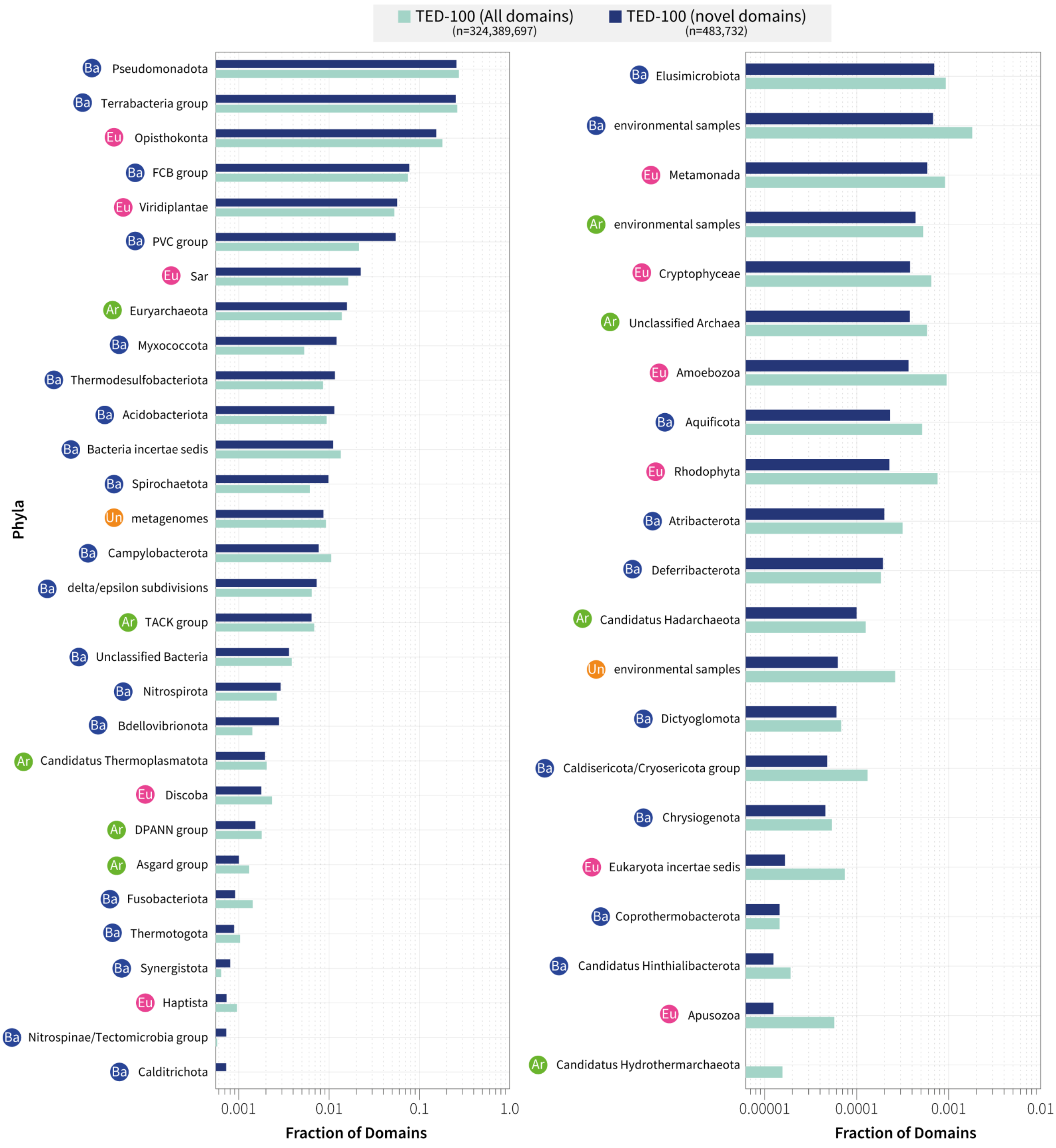

Fig. S12. Comparison of taxonomic distribution of identified novel domains against baseline proportions in TED-100. Bar charts compare the fraction of domains found in each phyla for novel domains (navy) and all domains in TED-100 (light blue). Phyla are ranked according to the novel domain fraction found. Labels next to each phylum denote the superkingdom: bacteria (Ba), eukarya (Eu), archaea (Ar) and unclassified (Un).

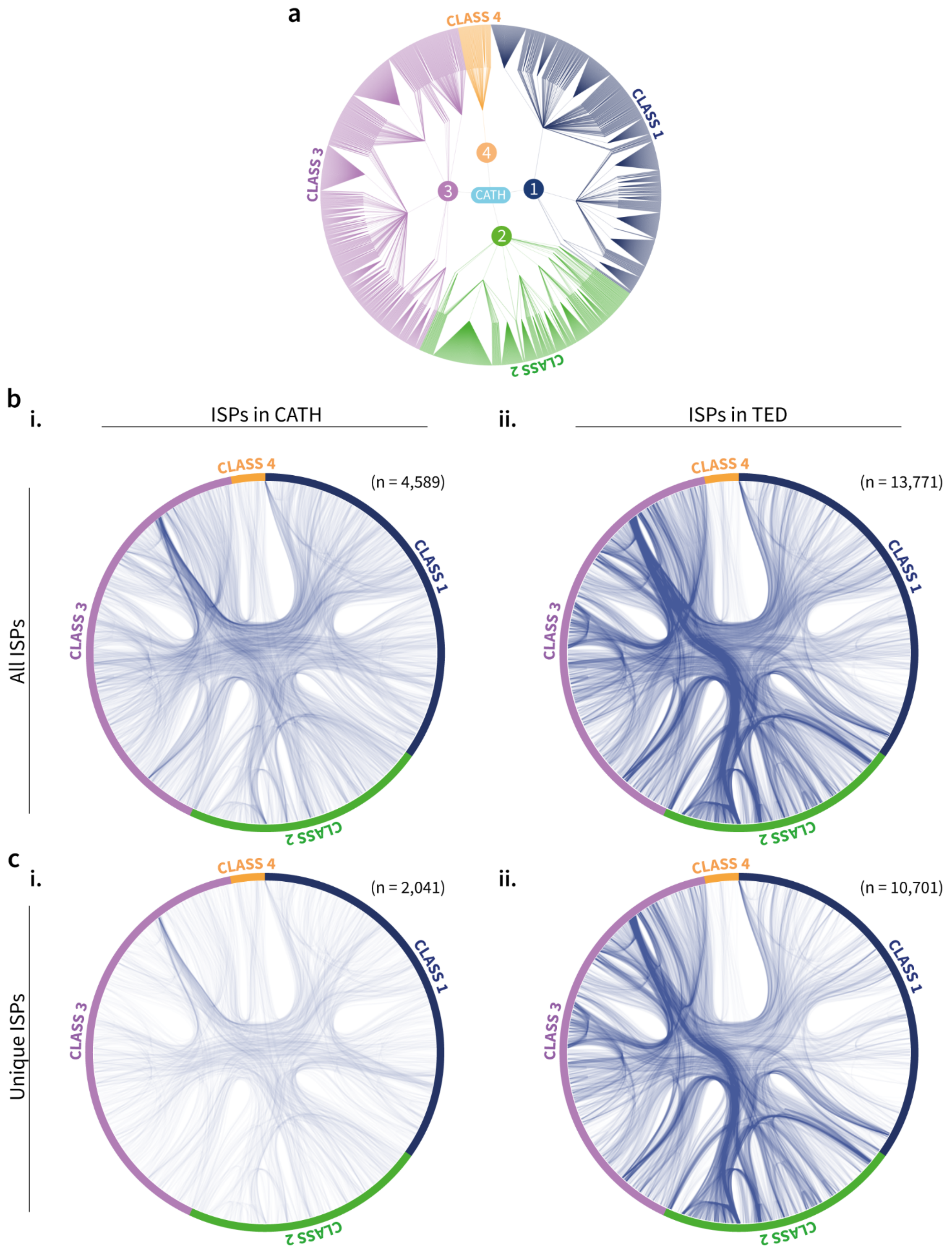

Fig. S13. (**a**). The CATH hierarchy (classes 1-4 only) is represented as a circular dendrogram with a virtual root node for the 4 classes (labelled 'CATH'). Edges are coloured according to CATH class. Paths drawn in the hierarchical edge bundling diagrams in (b) and (c) follow the layout of this dendrogram smoothly when connecting superfamilies, which are the leaf nodes on the outer edge. Hierarchical edge bundling diagrams representing (**b**) all ISPs and (**c**) ISPs unique to either set, for i. CATH, and ii. TED.

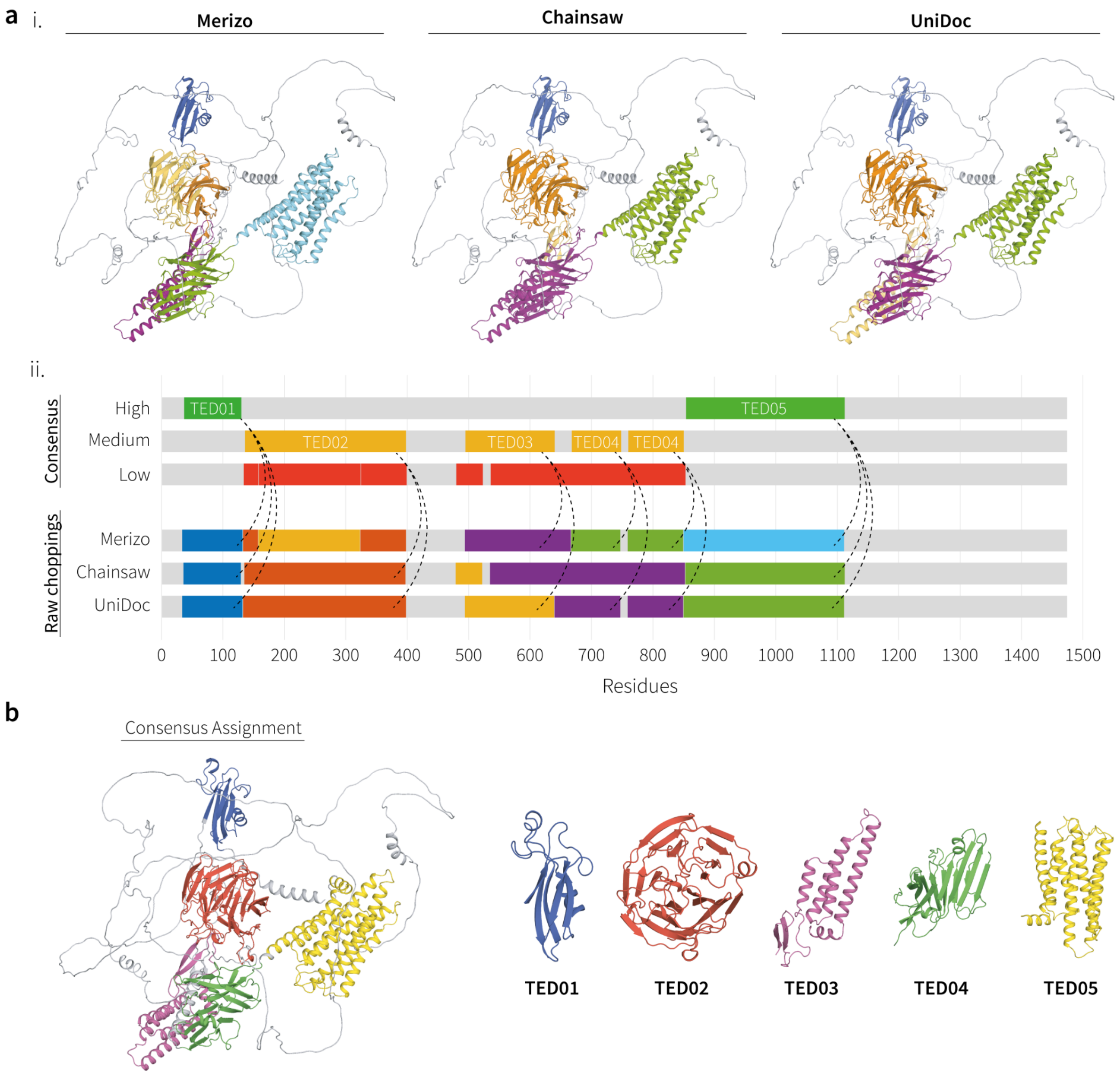

Fig. S14. Example of consensus domain derivation. (**a**) i. Example of predicted domain choppings from Merizo, Chainsaw and UniDoc for target AF-O94910-F1-model_v4 (Adhesion G protein-coupled receptor L1 from *Homo sapiens*). Predicted domains are shown in alternating colours. ii. Domain ranges in panel i. shown in one dimension along with the consensus classification for predictions across the three methods. High and medium consensus domains are those where three and two predictions from constituent methods agree. Low consensus domains are any single-method only predictions with low support and are not part of TED-100. Agreement between domain ranges are calculated based on a minimum of 70% sequence overlap between multiple domain ranges. Dashed lines have been drawn on the consensus panel to indicate source ranges used to derive the consensus from for medium and high confidence categories. TED domains are named sequentially beginning from the N-terminus. (**b**) Example of the consensus assignment for target AF-O94910-F1-model_v4 derived in panel (a), alongside views of individual identified domains. Domains are shown in alternating colours.

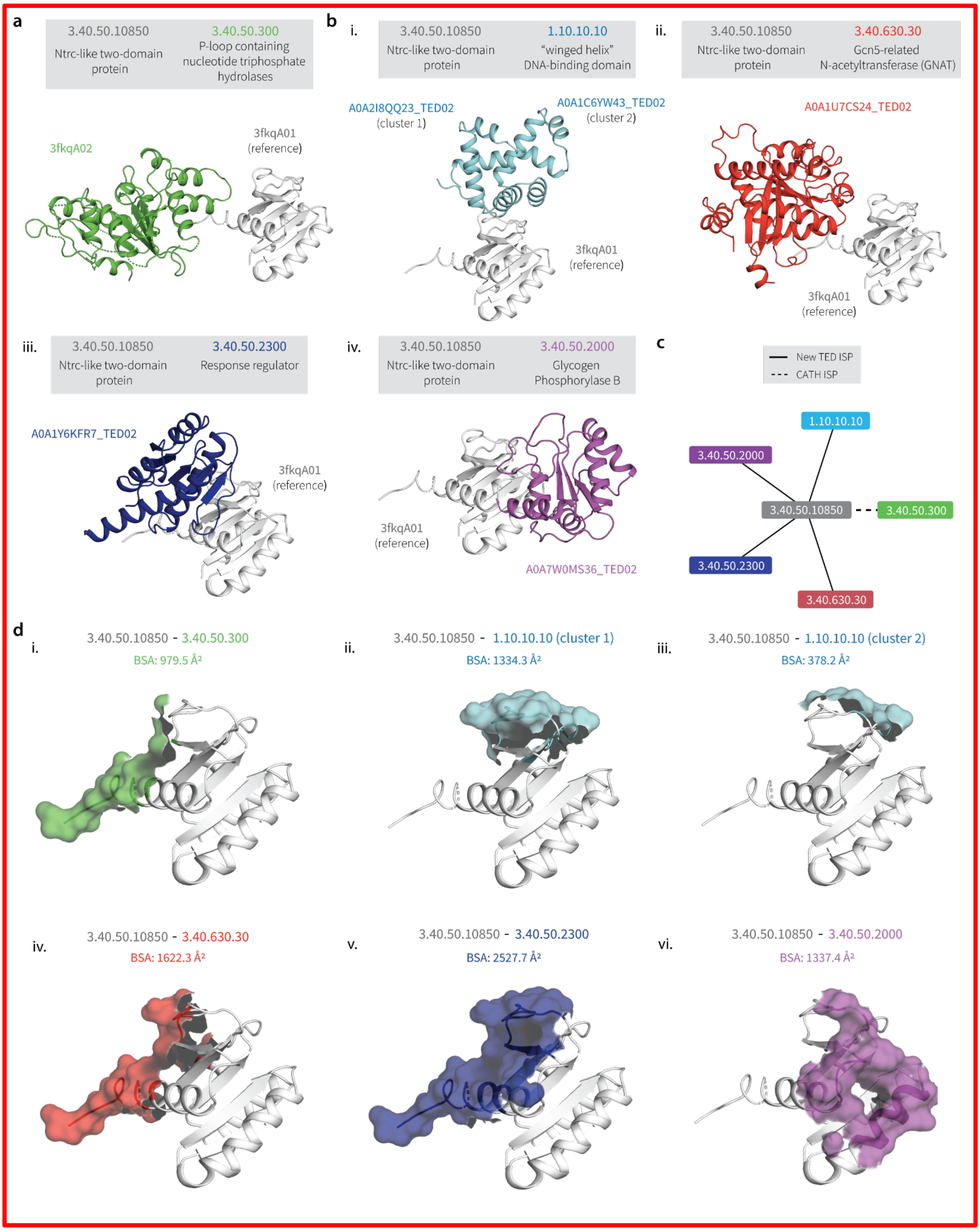

Fig. S15. Interactions for CATH superfamily 3.40.50.10850. (**a**) In the PDB, the NtrC-like protein domain (superfamily 3.40.50.10850) is only found interacting with a single P-loop-containing nucleotide triphosphate hydrolase family (superfamily 3.40.50.300). (**b**) In TED, superfamily 3.40.50.10850 can be seen interacting with four additional CATH superfamilies - i. 1.10.10.10 ("winged-helix" DNA-binding domain), ii. 3.40.630.30 (Gcn5-related N-acetylransferase), iii. 3.40.50.2300 (response regulator) and iii. 3.40.50.2000 (glycogen phosphorylase B) superfamilies. (**c**) Network graph showing existing interactions seen in

CATH (PDB) and new interactions observed in TED for superfamily 3.40.50.10850. (**d**) Surface patches corresponding to domain-domain interfaces are shown for each pair of superfamilies in panel (a). The buried surface area (BSA) is shown for each panel and was calculated using DSSP (*57*).

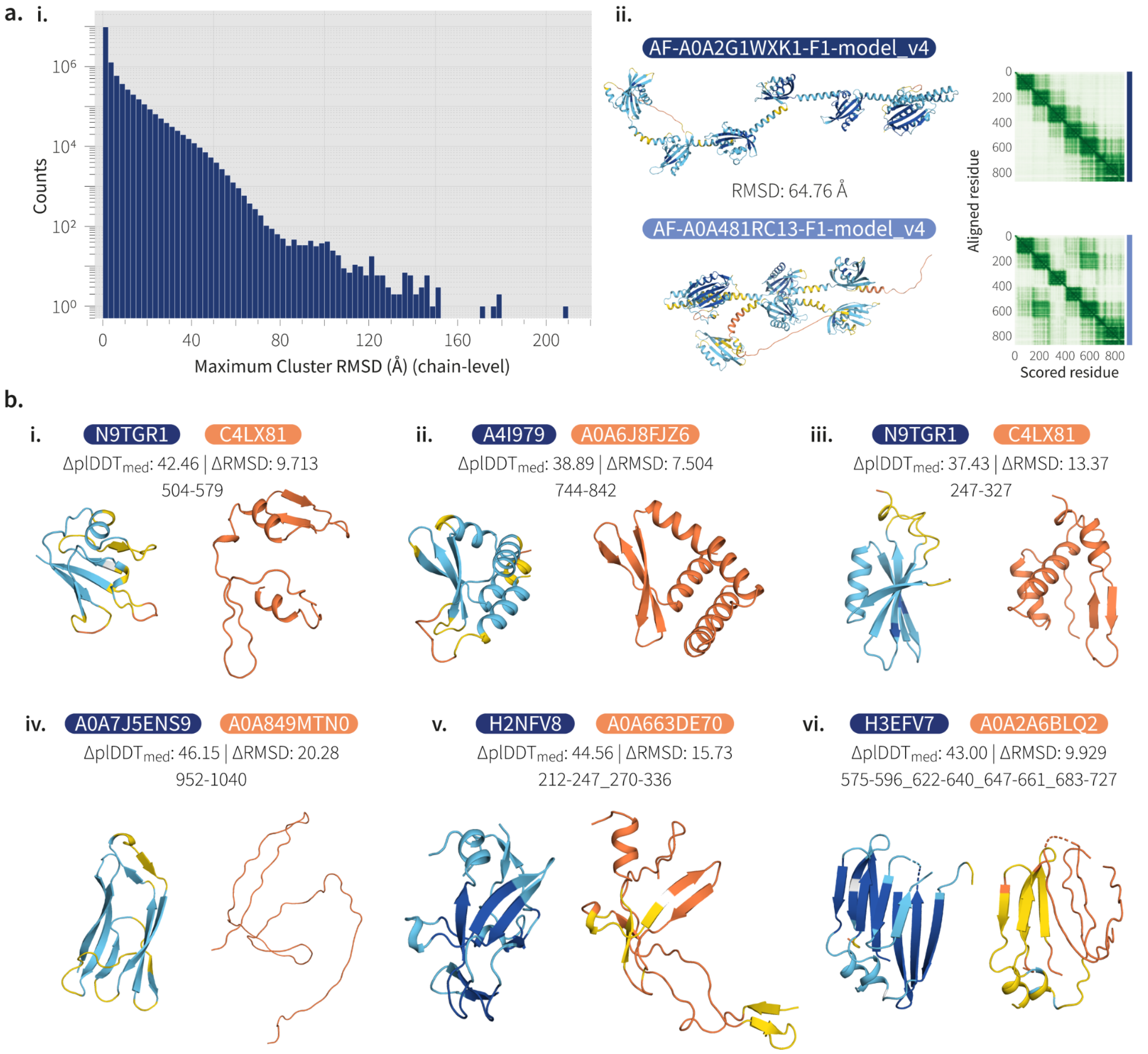

Fig. S16. Structural diversity across identical sequences in the AFDB. (**a**) i. Distribution of maximum RMSD observed across structures of identical sequences (n=13,175,417). For each identical sequence cluster, the maximum RMSD observed across pairs of structures is reported. Dataset comprises all TED-redundant targets (38,944,835 chains across 13,175,417 unique sequences). ii. Example of a pair of structures with identical sequences (full chain). Dark areas in PAE maps represent high confidence in the positioning between pairs of residues. Differences can be seen in domain packing as well as in the PAE map produced by AF2. AF2 has greater confidence in the packing of domains 2 and 5 in the bottom structure compared to the top. (**b**) i-vi. Examples of structural diversity in domains of identical sequence models. In each example, the difference in median domain plDDT as well as RMSD following TMalign is shown. Domain structures represent the exact same residue ranges in two models with identical full chain sequences. Colouration follows plDDT confidence bins as per the AFDB (dark blue/very high: plDDT >= 90, blue/high: 90 > plDDT >= 70, yellow/low: 70 > plDDT >= 50 and orange/very low: plDDT < 50).

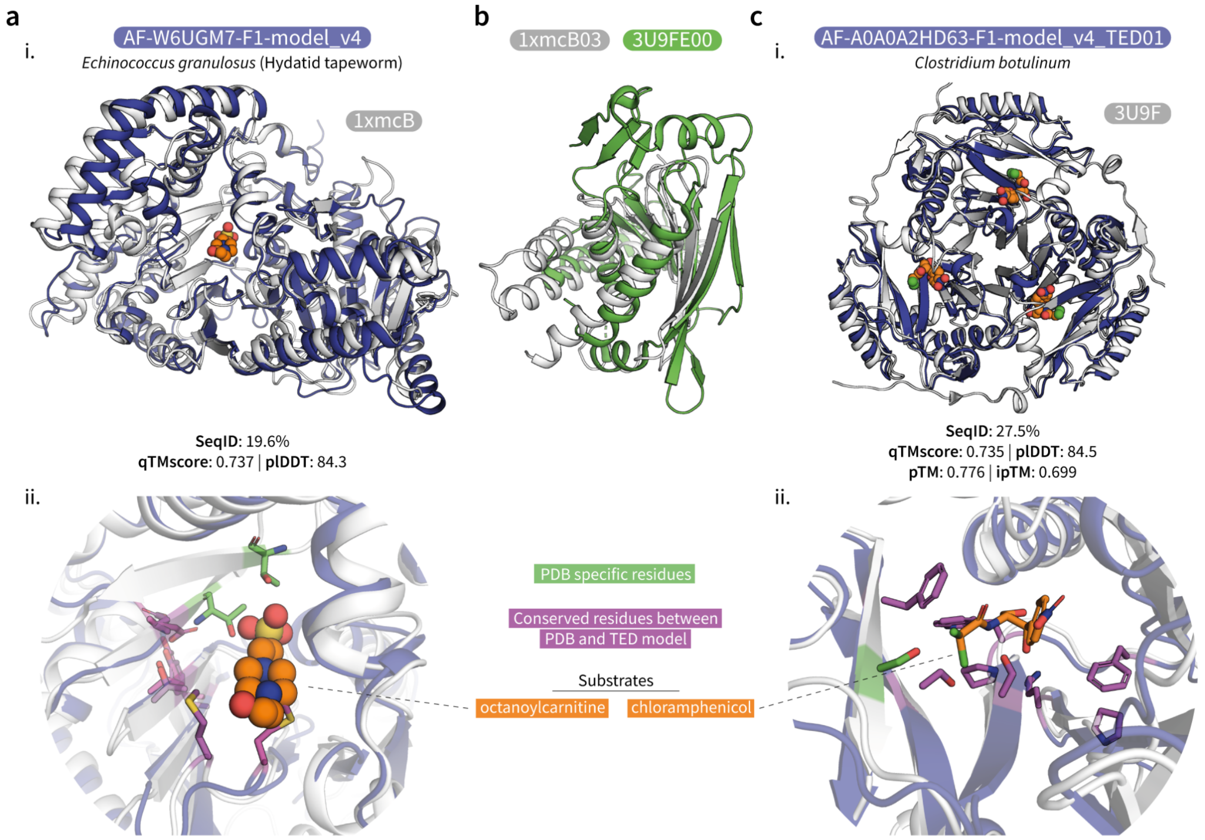

Fig. S17. Structural insights into differences and commonalities among CoA-dependent acyltransferases. (**a**) i. Superposition of AF-W6UGM7-F1-model_v4 and 1xmcB. ii. Substrate binding pocket of (a) with substrate (orange), conserved residues unique to PDB:1xmc (green) and shared between PDB:1xmc and TED monomer (magenta). (**b**) Superposition of CATH structural domain representatives clustered at 5Å for homotrimers (CATH: 3u9FE00) and monomers (1xmCB03). (**c**) i. Superposition of 3u9F and a modelled trimer of AF-A0A0A2HD63-F1-model_v4_TED01. ii. Substrate binding pocket of (c) with substrate (orange), conserved residues unique to PDB:3u9F (green) and shared between 3u9F and TED trimer (magenta).

Present in most taxonomic branches of the Tree of Life, the chloramphenicol acetyltransferase-like domain superfamily in CATH (3.30.559.10) adopts an αβα sandwich fold. The superfamily comprises a wide range of enzymes that function as CoA-dependent acyltransferases, with chloramphenicol acetyltransferases among others. Members of the superfamily share a conserved HXXXD motif in their active site and their oligomerization state can vary dramatically, from the more preponderant 2-domain single monomer **(a)**, to a more complex homo-trimer (**c**). Their oligomeric modes are fundamental to their catalytic functions. For example, carnitine octanoyltransferase functions as a monomer and comprises a N-terminal and a C-terminal domain which together form a substrate binding pocket [PDB ID: 1xmc, **a**], whereas chloramphenicol acetyltransferases adopt a homo-trimeric assembly for substrate-binding and catalytic activity (*58*) [PDB: 3u9F, **c**]. Ubiquitous and with a known biochemistry, chloramphenicol acetyltransferases play a critical role in antibiotic resistance in pathogens and

are prime targets for drug repurposing aimed at untreated pathogens of interest in health and agriculture.

TED vastly expands the structural coverage of the superfamily, from 253 domains in CATH to 228,867 in TED (Supp. Methods), aiding knowledge transfer of conserved residues and oligomerization states between remote homologs. Low sequence identity can be bridged by structure, enabling the identification of very remote homologs across TED using matching by structure instead of sequence. In panels a) and d), a remote homolog (AF-W6UGM7-F1-model_v4_TED03; Carnitine O-palmitoyltransferase; EC 2.3.1.21) of mouse carnitine octanoyltransferase (PDB:1xmcB003; carnitine octanoyltransferase; EC: 2.3.1.137) can be detected in hydatid tapeworm (qTMscore=0.737), a domestic pathogen by structure matching despite having a low sequence similarity (19.6%). By clustering highly structurally similar TED relatives (Supp. Methods), we could predict highly conserved residues for the tapeworm domain. The majority of these occur in a pocket which superimposes with the known substrate binding region in the experimental structure and furthermore the majority of the predicted conserved residues in the tapeworm domain superimpose well with those in the mouse domain. Despite this, structural analysis shows that the PDB structure has an insertion of two additional beta strands in the substrate binding pocket, which possess conserved residues unique to the PDB domain (shown in blue in panel a-ii)) suggesting a role in the distinct substrate specificity (2.3.1.137 rather than EC 2.3.1.21) of this enzyme.

Panel c) show superposition of a structural homolog in AFDB (AF-A0A0A2HD63-F1-model_v4_TED01), of a known chloramphenicol acetyltransferase (3u9F in the PDB). The AFDB homolog is from *Clostridium botulinum*, a pathogenic bacteria causing botulism in humans and animals. Whilst the sequence similarity is below 30%, the TED domain matches the PDB with a TM-Score 0.82. Following modelling of the trimeric oligomer of this protein we observe a good superposition with the trimer of PDB structure. We also see that highly conserved residues in the TED domain (identified from related structures in the AFDB) superimpose well with conserved residues in the PDB domain in the Chloramphenicol-binding site (see panel c-ii), supporting similar substrate binding.

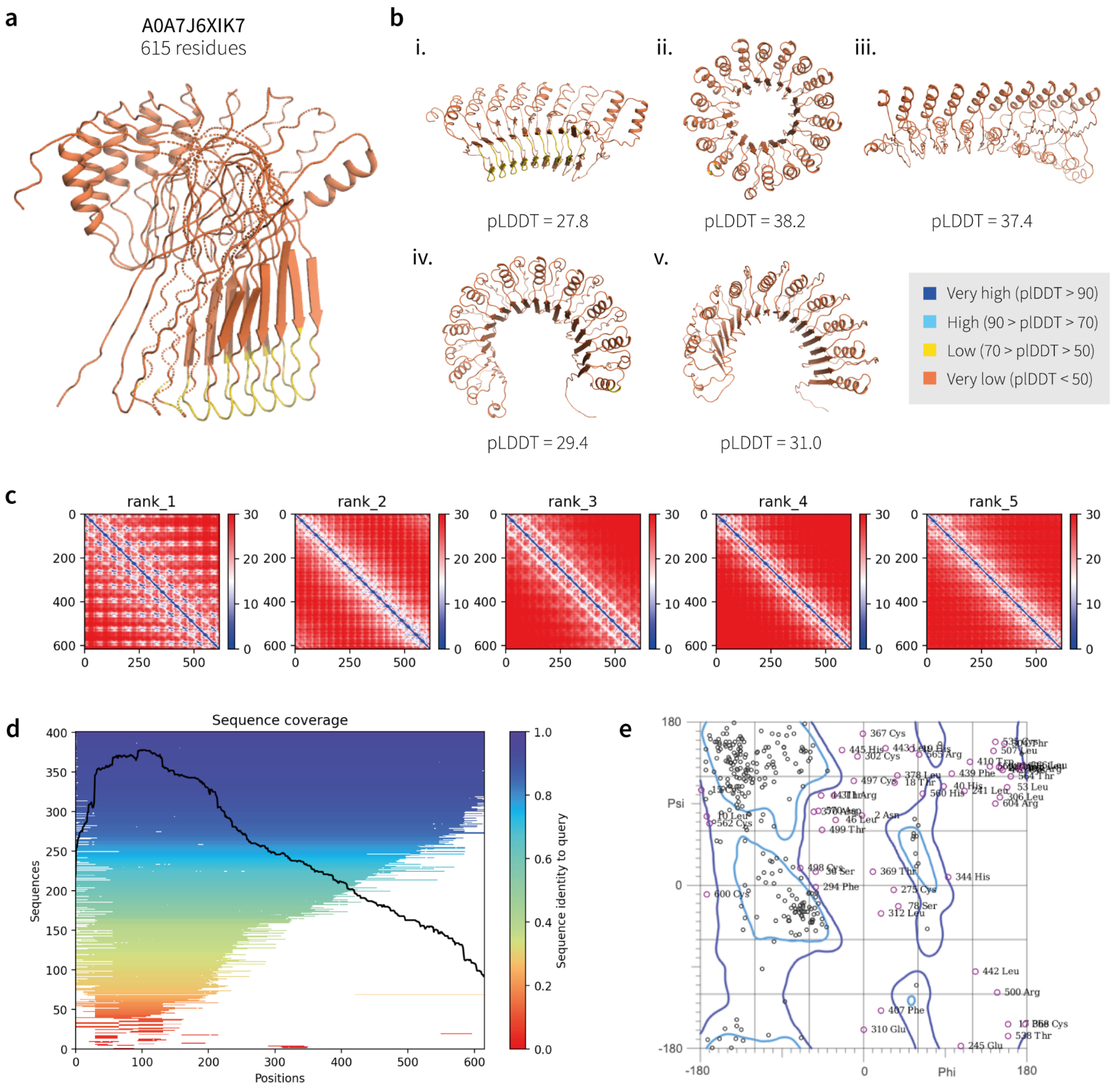

Fig. S18. Example of a problematic structure in the AFDB. (**a**) The AFDB model of Uniprot entry A0A7J6XIK7 from *Trypanosoma cruzi* is a low plDDT structure with a high proportion of clashes. (**b**) Re-prediction of the structure using Colabfold generates several visually plausible structures with high symmetry and repetition. All models are predicted with very low plDDT. (**c**) PAE output for each of the five structures is shown in panel (b). Blue colouration indicates high confidence between the position of residue pairs. (**d**) Sequence coverage of the MSA generated during remodelling in (b). (**e**) Molprobity Ramachandran plot of model shown in (a). Text-labelled residues have torsion angles outside of allowed regions.

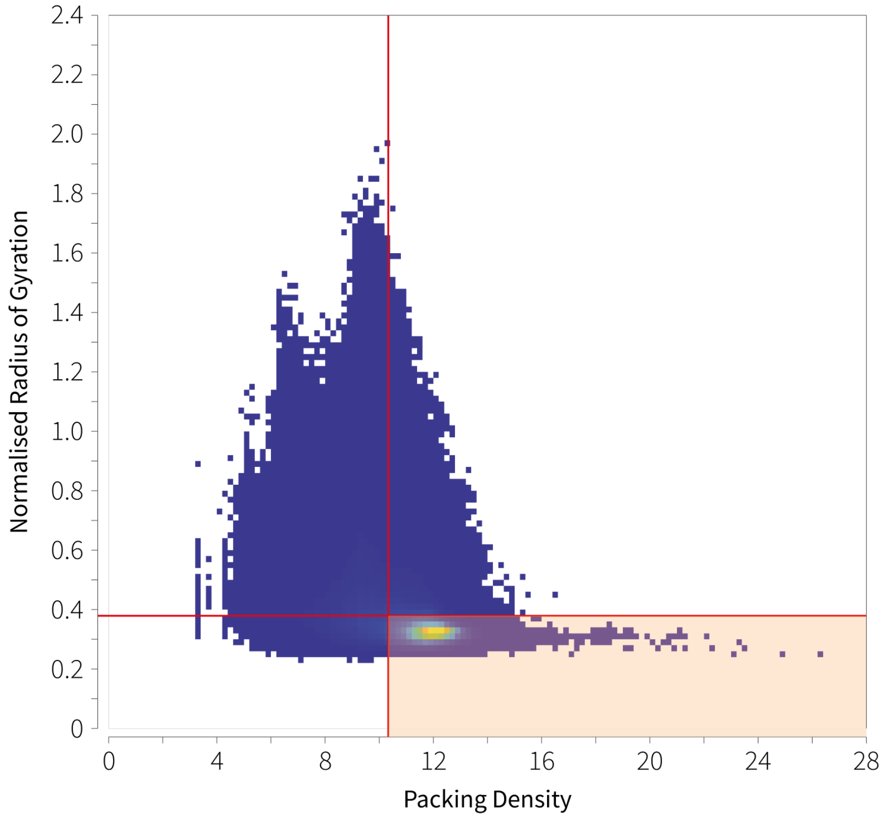

Fig. S19. Globularity assessment of domains in TED-100. Globularity of 324m TED-100 domains were assessed with CATH-AlphaFlow using packing density and normalised Rg metrics as described in Bordin et al. (*6*). To determine suitable cutoffs for identifying globular domains, thresholds were calculated as the 5th percentile of each metric on the subset of TED-100 domains which were assigned high-confidence CATH superfamily labels (193m domains). Packing density and normalised Rg cutoffs were 10.333 (vertical red line) and 0.356 (horizontal red line) respectively. Colour gradient on bivariate histogram represents the density of data, where yellow is highest, and navy is lowest. Shaded red box encapsulates 244,166,721 domains deemed globular.

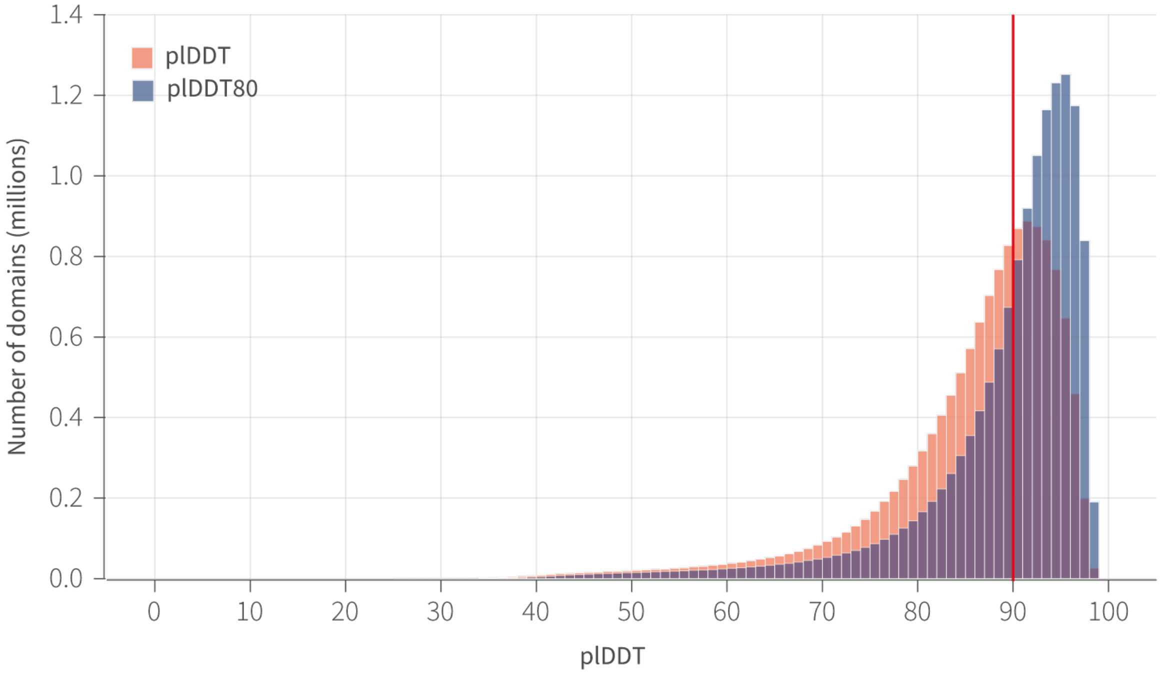

Fig. S20. Comparison of plDDT metrics for model quality filtering. Histograms shown represent the plDDT and $plDDT_{80}$ metrics used for model quality filtering. Data comprises 13,820,550 domains which pass globularity and nSSE filters. plDDT is calculated as the average plDDT of all residues in the domain while $plDDT_{80}$ takes into account the average residue plDDT over the highest 80% of residues. A $plDDT_{80}$ >= 90 is used for filtering domains before the novel domain identification workflow, resulting in 8,612,318 domains.

Supplementary Tables

Table S1. Breakdown of TED workflow.

**Step**	**No. targets**	**No. Clusters**
**i. Datasets**		
No. targets (full AFDB)	214,683,829	188,914,411
TED-100	188,914,411	
TED-redundant	38,944,835	
	**No. domains**	**No. Clusters**
**ii. Domain assignment (TED-100)**		
Raw domains (Merizo)	400,444,974	
Raw domains (Chainsaw)	328,956,414	
Raw domains (UniDoc)	366,117,430	
Medium consensus	129,160,426	
High consensus	250,629,037	
Total no. domains (TED-100)	324,389,697	
**iii. Classification**		
Superfamily labels assigned	193,939,494	
Topology labels assigned	45,796,122	
Domains with no labels	84,654,081	
Sequence clusters (50% identity, 90% coverage)	324,389,697	120,748,700
Non-singletons	242,723,300	39,082,303
with CATH label	202,318,609	29,872,777
without CATH label	40,404,691	9,209,526
Singletons	81,666,397	81,666,397
with CATH label	48,996,065	48,996,065
without CATH label	32,670,332	32,670,332

Total CATH assignable	251,314,674	78,868,842
Annotations transferrable	11,579,058	
Total unassigned	73,075,023	41,879,858
Singletons	32,670,332	32,670,332
Non-singletons	40,404,691	9,209,526
Passing globularity, nSSE, plDDT filters	19,816,697	8,612,318
Singletons	5,888,040	5,888,040
Non-singletons	13,928,657	2,724,278
Discarded by filters	53,258,326	33,267,540
Singletons	26,782,292	26,782,292
Non-singletons	26,476,034	6,485,248
**iv. Novel fold workflow**		
Structures considered	19,816,697	8,612,318
Structure clusters	8,612,318	625,802
Clusters matched to PDB, ECOD, SCOPe and CATH	13,297,059	385,128
Unmatched clusters	6,519,638	240,674
High internal symmetry clusters	277,694	6,433
Putative novel clusters (low symmetry)	3,614,884	138,120
Final novel clusters (after final searches)	483,732	7,427
Singletons	1,930	1,930
Non-singletons	481,802	5,497

Table S2. Topology and superfamily names of CATH codes shown in Fig. 2**.**

CATH code	Topology Name	Superfamily Name
1.20.120.1730	Four Helix Bundle (Hemerythrin (Met), subunit A)	-
1.10.3980.10	T-fold	ApbE-like superfamily
1.10.150.170	DNA polymerase; domain 1	Putative methyltransferase TM0872, insert domain
1.10.1740.110	Rna Polymerase Sigma Factor; Chain: A	-
1.25.40.890	Serine Threonine Protein Phosphatase 5, Tetratricopeptide repeat	-
2.40.50.250	OB fold (Dihydrolipoamide Acetyltransferase, E2P)	bipa protein
2.60.40.2250	Immunoglobulin-like	-
2.60.120.810	Jelly Rolls	-
2.70.20.10	Topoisomerase I; domain 3	Topoisomerase I, domain 3
2.60.40.3450	Immunoglobulin-like	-
3.30.70.1020	Alpha-Beta Plaits	Trehalose-6-phosphate phosphatase related protein; domain 2
3.30.70.2750	Alpha-Beta Plaits	-
3.10.450.630	Nuclear Transport Factor 2; Chain: A	-
3.30.70.1910	Alpha-Beta Plaits	-

Table S3. Comparison of coverage between Bordin et al., 2023 and TED for AFDB 21 model organisms dataset.

Source	AF21	AF21 (%)	TED21	TED21 (%)
Chopped domains	708,941		1,300,686	
Domains over 70 plDDT	532,412	75.04%	1,176,636	90,46%
Good quality domains	369,512	52.08%	867,174	66,67%
Good quality domains in CATH	341,213	92.3%	718,703	82.87%

The characterisation of the initial release of the AFDB, described in Bordin et al. (*6*), increased the structural coverage for 21 model organisms with over 341,000 domains with good quality assigned to CATH. Using the same thresholds specified in the original article on TED domains associated to proteomes from the original release, we noticed how TED nearly doubles the number of chopped domains, and its consensus approach identifies a higher proportion of domains with high quality (AF21=52%, TED21=67%). The percentage of good quality domains assigned to CATH with TED is slightly lower whilst the total count is higher than AF21, suggesting that the original AF21 dataset comprised mostly close homologs, while TED encompasses those and further globular domains with more remote relationships.

Table S4. Top 20 most frequent GO molecular function terms predicted in the set of domains with novel folds.

GO term	Count	Description
GO:0016491	386	oxidoreductase activity
GO:0016301	120	kinase activity
GO:0008270	110	zinc ion binding
GO:0008168	101	methyltransferase activity
GO:0003676	87	nucleic acid binding
GO:0008233	80	peptidase activity
GO:0020037	72	heme binding
GO:0016874	53	ligase activity
GO:0004519	43	endonuclease activity
GO:0016829	39	lyase activity
GO:0008237	25	metallopeptidase activity
GO:0009055	25	electron transfer activity
GO:0004222	23	metalloendopeptidase activity
GO:0016757	23	glycosyltransferase activity
GO:0051536	22	iron-sulphur cluster binding
GO:0005509	21	calcium ion binding
GO:0022857	21	transmembrane transporter activity
GO:0003723	20	RNA binding
GO:0003968	19	RNA-dependent RNA polymerase activity

Table S5. Top 20 most frequent GO molecular function terms predicted in the set of novel repeat domains.

GO term	Count	Description
GO:0016491	278	oxidoreductase activity
GO:0016301	127	kinase activity
GO:0022857	89	transmembrane transporter activity
GO:0008270	87	zinc ion binding
GO:0003677	79	DNA binding
GO:0003700	77	DNA-binding transcription factor activity
GO:0003676	76	nucleic acid binding
GO:0016829	74	lyase activity
GO:0008233	59	peptidase activity
GO:0008168	56	methyltransferase activity
GO:0005509	47	calcium ion binding
GO:0016874	43	ligase activity
GO:0004650	36	polygalacturonase activity
GO:0020037	36	heme binding
GO:0043565	34	sequence-specific DNA binding
GO:0016757	27	glycosyltransferase activity
GO:0030246	26	carbohydrate binding
GO:0009055	25	electron transfer activity
GO:0016853	24	isomerase activity
GO:0046872	19	metal ion binding
